# Variations of Major Flavonoids, Nutritional Components, and Antioxidant Activities in Mung Beans (*Vigna radiate* L.) of Different Seed Weights

**DOI:** 10.3390/foods13213387

**Published:** 2024-10-24

**Authors:** Kebede Taye Desta, Yu-Mi Choi, Jungyoon Yi, Myoung-Jae Shin, Young-ah Jeon, Hyemyeong Yoon

**Affiliations:** 1 National Agrobiodiversity Center, National Institute of Agricultural Sciences, Rural Development Administration, Jeonju 54874, Republic of Korea; kebedetdesta@korea.kr (K.T.D.); cym0421@korea.kr (Y.-M.C.); naaeskr@korea.kr (J.Y.); smj1204@korea.kr (M.-J.S.); yjeon@korea.kr (Y.-a.J.); 2 Department of Applied Chemistry, College of Natural and Computational Sciences, Adama Science and Technology University, Adama P.O. Box 1888, Ethiopia

**Keywords:** antioxidants, biochemicals, fatty acids, flavonoids, legume, nutrition, seed weight

## Abstract

This study examined the levels of major flavonoids, nutritional components, total secondary metabolite contents, and antioxidant activities in 136 mung bean accessions and statistically analyzed the effect of seed weight difference on each. Vitexin and isovitexin were detected in all the mung bean accessions, with isovitexin being in a higher concentration regardless of seed weight difference. The contents of total protein and total starch were in the ranges of 22.01–28.96 and 32.62–49.03 g/100 g, respectively. Five fatty acids were detected by GC–FID analysis in all mung bean accessions, with linoleic acid being the most dominant (37.96–50.71 g/100 g). Total saponin content (TSC), total phenol content (TPC), DPPH^•^ scavenging activity, ABTS^•+^ scavenging activity, and ferric reducing antioxidant power (FRAP) showed more than five-fold differences. Analysis of variance supported by multivariate analysis demonstrated that seed weight difference had a significant effect on total starch, all individual fatty acids except for stearic acid and oleic acid, TSC, and all antioxidant activities except for ABTS^•+^ scavenging activity. On the other hand, vitexin, isovitexin, total protein, total phenol, and total fatty acid contents remained unaffected by seed weight difference. Overall, this study showed the diversity of key flavonoids, nutritional components, total secondary metabolite contents, and antioxidant activities in mung bean genetic materials. Moreover, the study unveiled how seed weight affects the analyzed parameters in mung beans for the first time. These findings could maximize the use of mung beans in food industries and breeding programs as well as lead to more studies in metabolomics and genomics.

## 1. Introduction

Mung bean (*Vigna radiate* L.) is one of the most popular legumes worldwide [1,2,3]. It is believed that mung bean originated in India, but it is currently grown in various parts of the world including Asia, Africa, and America [4,5]. Mung bean seeds contain various beneficial metabolites that promote human health. These include nutritional components such as protein, amino acids, starch, vitamins, fatty acids, and minerals. These qualities make mung beans important sources of energy and crucial components in the diets of numerous populations [5,6,7].

In addition to their nutritional values, mung beans also contain several bioactive natural compounds such as flavonoids, anthocyanins, and phenolic acids. These polyphenols are known to have antioxidant and anti-inflammatory properties and, hence, are responsible for the health-promoting properties of mung beans [8,9,10]. Vitexin and isovitexin are recognized as the primary flavonoids found in mung beans. These isomeric and C-glycosylated compounds have been shown to effectively reduce oxidative stress and lower the risk of developing different ailments such as cancer, heart disease, and diabetes [11,12,13]. A recent review also highlighted the neuroprotective benefits of mung bean polyphenols and their role in preventing Alzheimer’s disease [14]. Due to these desirable qualities, there has been a growing interest in exploring the diversity of mung bean genetic materials in recent years [15,16]. On the other hand, several environmental and genetic factors can affect the overall chemical composition of dietary crops in general [17,18,19]. These environmental factors include differences in growth location, soil type, climate, and years of cultivation, while genetic factors include diseases, pests, genotype variations, and seed-related traits such as seed color and seed weight [19,20,21]. Seed weight is one of the most important traits for optimizing yield and, hence, widely used in legume breeding. It is also a heritable trait and can vary within and among varieties influencing the levels of bioactive compounds present [21,22].

Previously, several studies have explored the effects of various environmental and genetic factors on the levels of health-promoting secondary metabolites and nutritional components in different crop genetic materials [1,23,24]. Understanding the effects of these factors has led to the development of improved legume varieties that can withstand different adverse conditions while also enhancing their nutritional values and health advantages [25,26,27]. Compared to other legumes, however, there has been limited research conducted on mung beans. One possible reason for the lack of such studies could be the limited availability of a large collection of mung bean genetic resources compared to other legumes [16]. Accordingly, this study aimed to investigate the diversity of major flavonoids, nutritional components, total secondary metabolite, and antioxidant activities in a large collection of mung bean genetic materials and analyze the effect of seed weight difference on each. Specifically, a total of 136 mung bean accessions were cultivated, collected, and categorized based on seed weight (small, medium, and large). Then, statistical analysis was used to investigate the diversity as well as the effect of seed weight on the levels of flavonoids (including vitexin and isovitexin), total protein content, total starch content, five distinct fatty acids (including palmitic acid, stearic acid, oleic acid, linoleic acid, and linolenic acid), total phenol content, total saponin content, and antioxidant activities (1,1-Diphenyl-2-picrylhydrazyl radical (DPPH^•^) scavenging activity, 2,2′-azino-bis(3-ethylbenzothiazoline-6-sulfonic acid) diammonium radical cation (ABTS^•+^) scavenging activity, and ferric reducing antioxidant power (FRAP)). To the best of our knowledge, this study is the first to unveil how seed weight affects the analyzed parameters in mung beans. Overall, the study may offer valuable information regarding the associations of seed weight with the nutritional and secondary metabolite components of mung beans, which could be applicable in food industries and mung bean breeding programs.

## 2. Materials and Methods

### 2.1. Chemicals and Reagents

The chemicals and reagents used in this study were of analytical grade. Sulfuric acid and ethanol were ordered from Fisher Scientific (Pittsburgh, PA, USA). The other chemicals and reagents, such as methanol, vitexin, isovitexin, fatty acid standards (palmitic acid, stearic acid, oleic acid, linoleic acid, and linolenic acid), gallic acid, formic acid, L-ascorbic acid, anhydrous sodium carbonate (Na_2_CO_3_), potassium ferricyanide, trichloroacetic acid, vanillin, ferric chloride, 1,1-diphenyl-2-picrylhydrazyl (DPPH) radical, 2,2′-azino-bis(3-ethylbenzothiazoline-6-sulfonic acid) diammonium salt (ABTS), and Folin–Ciocalteu phenol reagent, were purchased from Sigma-Aldrich (St. Louis, MO, USA).

### 2.2. Seed Material Collection, Cultivation, and Preparation

The seeds of the 136 mung bean accessions were obtained from the gene bank at the National Agrobiodiversity Center, Rural Development Administration (RDA, Jeonju, Republic of Korea). These accessions were cultivated in an experimental field at the National Agrobiodiversity Center (Jeonju, Latitude/Longitude: 30°49′38.37″ N/127°09′7.78″ E) from June 2022 to October 2022 under uniform growth conditions. For each accession, 30 seeds were sown on clay loam soil in 15 cm apart rows. The distance between seeds was maintained at 90 cm. During the cultivation time, the average temperature was 23.7 °C in June, 27.0 °C in July, 26.3 °C in August, 22.6 °C in September, and 15.2 °C in October. Likewise, the average precipitation was 164.2, 184.0, 311.3, 64.5, and 51.1 mm, while the average humidity was 73.0, 78.0, 79.0, 70.0, and 67.0% in June, July, August, September, and October, respectively. Matured seeds were manually harvested. The one-thousand seed weight of each accession was determined from randomly selected triplicate measurements, and the mung bean accessions were categorized as small (<50 g 1000^−1^ seeds, n = 87), medium (50–60 g 1000^−1^ seeds, n = 14), and large (>60 g 1000^−1^ seeds, n = 35) seeds [28]. Then, samples from each category, in triplicate, were dried, powdered, and stored in sealed plastic bags at −20 °C pending further analysis. General information regarding all the mung bean accessions including their introduction (IT) number, code given (MB1-MB136), and seed weight, among others, is provided in Table S1 (Supplementary Material).

### 2.3. Extraction and Analysis of Flavonoids Using RP-HPLC–DAD Analysis

Flavonoid extraction was conducted using a previously reported protocol with some modifications [29]. Initially, 0.5 g of powdered mung bean sample was mixed with 50 mL of 80% ethanol and sonicated for 1 h in a water bath set at 30 °C. The mixture was then taken off and centrifuged (3134× *g*) at 25 °C for 15 min and then filtered, and the supernatant was retained. The residue was extracted for the second time using 2.5 mL of the extraction solvent. Then, the combined supernatant was filtered through a membrane syringe filter and stored at −20 °C until analysis. The identification and quantification of vitexin and isovitexin were accomplished using a reverse-phase 1260-Infinity quaternary high-performance liquid chromatography system (Agilent Technologies, Santa Clara, CA, USA) with a diode-array detector (RP-HPLC–DAD). A sample volume of 5 µL was injected, and separation was carried out using an Agilent Zorbax SB C-18 column (4.6 × 250 mm, 5 μm) maintained at 35 °C. The mobile phase was a binary solvent system consisting of water (A) and methanol (B), each with 0.2% acetic acid, at a flow rate of 1 mL/min. The gradient condition started with 20% of solvent B and gradually increased to 35% over 15 min, to 40% over 10 min, and to 90% over 20 min. Then, the mobile phase was re-equilibrated to 20% of solvent B over 10 min with a post-run of 10 min. ChemStation software LTS 01.11 (Agilent Technologies, Santa Clara, CA, USA) was used to monitor the acquired chromatogram at 280 nm wavelength (λ). Vitexin and isovitexin were identified by comparing their retention times with corresponding standards and analyzing the UV–vis absorption spectra. Quantification was conducted by plotting calibration curves based on the peak area responses of vitexin and isovitexin external standards at concentrations ranging from 0.01 to 1.00 mg/mL. Then, the concentrations of vitexin and isovitexin in each mung bean accession were reported as milligrams per gram of dried seed weight (mg/g dw) from triplicate measurements.

### 2.4. Determination of Total Protein and Total Starch Contents

The total protein content was determined using the Kjeldahl method as detailed in a previous study [30]. Briefly, 0.5 g of powdered sample was mixed with 12 mL of concentrated H_2_SO_4_ and two pellets of selenium catalyst. The mixture was then digested in a Kjeltec instrument with an auto-digester (FOSS, Hillerød, Denmark) for 1 h to extract protein followed by cooling, distillation, and titration. The total protein content was then calculated by multiplying the released nitrogen content (N) by the standard conversion factor (6.25) and reported as g/100 g of dried seed weight. The total starch content was measured using a Megazyme Total Starch Assay Kit (Neogen Corporation, Wicklow, Ireland). The analysis was conducted utilizing the α-amylose/amyloglucoside assay protocol following the AOAC method 996.11.18 [31]. Absorbance was measured at λ_max_ of 510 nm using GENESYS 20 UV–Vis spectrophotometer (Thermo Scientific, Waltham, MA, USA), and the total starch content was reported as g/100 g of dried seed weight.

### 2.5. Extraction and Analysis of Fatty Acids Using GC–FID

Fatty acid methyl ester derivatives (FAMEs) were synthesized by a direct methylation method and used for fatty acid analysis [32]. Briefly, 0.2 g of powdered sample, 680 µL of a solvent mixture consisting of methanol, benzene, 2,2-dimethoxypropane, and sulfuric acid in the ratio of 39:20:5:2, and 400 µL of *n*-heptane were mixed in a 10 mL round-bottom glass tube with a screw cap. Then, the mixture was vortexed followed by extraction in a shaking water bath set at 80 °C. After 2 h, the mixture was then taken out, cooled at 25 °C, and centrifuged (3134× *g*, 15 min). The upper layer containing *n*-heptane with FAMEs was retained, filtered, and made ready for analysis. The identification and quantification of fatty acids was conducted using a QP2010 gas chromatography–flame ionization detector (GC–FID) instrument (Shimadzu, Kyoto, Japan). The column used for separation was an HP-INNOWAX column (30 m × 0.250 mm, 0.25 µm). The sample injection volume was 1 µL with a split ratio of 50:1. During analysis, helium was used as a carrier gas at a flow rate of 1.5 mL/min. The column temperature was initially set at 100 °C. Then, it was gradually increased to 170 °C at a rate of 60 °C/min and to 240 °C at a rate of 6.5 °C/min. In each case, the holding time was set at 1 min. The detector and injection port temperatures were maintained at 250 °C. LabSolution software (version 5.92, Shimadzu Kyoto, Japan) was utilized to process the acquired GC chromatograms. Fatty acids were identified based on the retention times of the corresponding external standards, and their contents were calculated as the percentage (g/100 g) of total fatty acids using area peak.

### 2.6. Determination of Total Saponin and Total Phenol Contents

The extraction protocol followed for vitexin and isovitexin analysis was also used for the determination of total saponin and total phenol contents [32]. The total phenolic content (TPC) was determined using the Folin–Ciocalteu method. Initially, 100 μL of the sample extract was combined with an equal amount of Folin–Ciocalteu phenol reagent. The mixture was then allowed to react in the dark at a temperature of 25 °C for 3 min. Following this, 100 μL of 2% sodium carbonate solution (Na_2_CO_3_) was added, and the mixture was incubated for an additional 30 min. The absorbance of the mixture was then measured at λmax of 750 nm using an Eon Microplate Spectrophotometer (Bio-Tek, Winooski, VT, USA). Then, TPC was expressed as milligrams of gallic acid equivalents per gram of dried seed weight (mg GAE/g) from triplicate measurements. The total saponins content (TSC) was determined using the vanillin–sulfuric acid method. Initially, 25 µL of the sample extract was combined with an equal amount of 8% vanillin in ethanol followed by the addition of 250 µL of 72% sulfuric acid in water. The mixture was then incubated in a water bath at 60 °C for 10 min followed by cooling in an ice bath for 15 min. Finally, the absorbance of the final mixture was measured at λ_max_ of 544 nm using an Eon Microplate Spectrophotometer (Bio-Tek, Winooski, VT, USA). The TSC was expressed in milligrams of diosgenin equivalent per gram of dried seed weight (mg DE/g) from triplicate measurements.

### 2.7. Determination of Antioxidant Activities

The same extraction method that was used to analyze vitexin and isovitexin was once again utilized for the determination of DPPH^•^ scavenging activity, ABTS^•+^ scavenging activity, and FRAP. The determination of each antioxidant activity was conducted according to our recently reported protocol without modification [32]. In summary, the levels of DPPH^•^ scavenging activity and FRAP were measured in milligrams of ascorbic acid equivalent per 100 g of dried seed weight (mg AAE/100 g), while the ABTS^•+^ scavenging activity was expressed in milligrams of Trolox equivalents per gram of dried seed weight (mg TE/100 g) from triplicate measurements.

### 2.8. Statistical Analysis

All measurements were conducted in triplicate unless stated otherwise, and the results were presented as the mean ± standard deviation (SD). Significant differences between measurements were determined by analysis of variance based on Duncan’s multiple range test computed at a 0.05 probability level using xlstat software version 2019.2.2 (Lumivero, CO, USA). Moreover, all the data obtained were auto-scaled and subjected to multivariate analysis. Principal component analysis (PCA) was conducted using JMP software version 17 (SAS, Inc., Cary, NC, USA). Hierarchical cluster analysis (HCA) and Pearson’s correlation matrix were computed by R software version 4.2.0 (http://www.r-project.org/) using ComplexHeatmap and Psych packages, respectively.

## 3. Results and Discussion

### 3.1. Variations of Vitexin and Isovitexin Contents

The distributions and contents of vitexin and isovitexin were determined by HPLC–DAD using the corresponding external standards (Figure S1). Both compounds were detected in all the studied mung bean accessions at different concentrations. The contents of vitexin and isovitexin in each of the 136 mung bean accessions are provided in Table S1, while summary data are presented in Table 1. In the whole population, the concentration of vitexin ranged from 0.21 to 1.33 mg/g dw with a mean of 0.50 mg/g dw, while isovitexin level ranged from 0.26 to 1.67 mg/g dw with a mean of 0.66 mg/g dw, both showing more than 6-fold variations. Among the studied accessions, MB110 had the highest levels of both vitexin and isovitexin. Conversely, accession MB130 had the lowest vitexin level, and accession MB39 had the lowest isovitexin level. In a previous study, vitexin content ranged from 0.44 to 1.50 mg/g, while isovitexin content ranged from 0.35 to 1.10 mg/g [33]. Another study found broader ranges of vitexin (17.04 to 62.37 mg/100 g) and isovitexin (22.63 to 73.64 mg/100 g) levels [34]. In contrast, Luo et al. [12] reported much higher concentrations of vitexin and isovitexin, reaching 7.47 and 7.51 mg/g, respectively. These findings demonstrate the variability in vitexin and isovitexin concentrations reported in different studies, which could be attributed to factors such as genetic variations, extraction techniques, growing conditions, the number of accessions analyzed, and postharvest practices, among others [34,35]. Overall, the vitexin and isovitexin contents found in this study were within previously reported ranges. Vitexin and isovitexin are known for their wide-ranging health benefits as highlighted before [13]. In this regard, those mung bean accessions containing higher levels of these compounds could be valuable resources.

### 3.2. Variations of Total Protein and Total Starch Contents

Standard procedures were used to determine the total protein and total starch levels in each of the 136 mung bean accessions. The total protein content ranged from 22.01 to 28.96 g/100 g with a coefficient of variation (CV) of 4.84%, while the total starch content ranged from 32.62 to 49.03 g/100 g with a CV of 7.37% in the whole population (Table S2 and Table 2). Accession MB56 had the highest protein content, while accession MB116 had the lowest. In contrast, accession MB7 had the highest total starch content, and accession MB68 had the lowest. Previously, Wang et al. [29] reported a total protein content of 17.36–24.89 g/100 g and a starch content of 39.54–60.66 g/100 g. In another study, Shi et al. [36] reported much narrower ranges of total protein (20.0–24.3%) and total starch (40.6–48.9%) contents compared to the results obtained in this study. Such wide-ranging results have also been reported by other studies, and the observed variations could once again be attributed to differences in environmental conditions, analysis methods, and genetic differences as highlighted before [19,37,38]. Protein and starch levels play a crucial role in determining the overall nutritional qualities of mung beans [39,40]. This study found that 14.71% of the mung bean accession had higher levels of both total protein and total starch contents than the average values (25.69 g/100 g and 40.85 g/100 g, respectively), suggesting that they could be valuable resources.

### 3.3. Variations of Fatty Acid Contents

This study also examined the distribution and levels of five major legume fatty acids using a GC–FID instrument. The levels of each fatty acid in all 136 mung bean accessions are provided in Table S2 and all showed significant variations (*p* < 0.0001). Palmitic acid and stearic acid, the two saturated fatty acids, ranged from 27.71 to 31.94 and 1.62 to 8.61 g/100 g, respectively (Table 2 and Table S2). Similarly, the unsaturated fatty acids including oleic acid, linoleic acid, and linolenic acid were found in the ranges of 1.62–3.15, 37.96–50.71, and 13.78–24.89 g/100 g, respectively. Total saturated fatty acid (TSFA) and total unsaturated fatty acid (TUFA) contents had values ranging from 31.47 to 37.68 and 62.32 to 68.53 g/100 g, respectively. Among the mung bean accessions, MB121 exhibited the highest TSFA and the lowest TUFA, while MB76 had the highest TUFA and TSFA. In general, the ranges found in this study were in agreement with previously reported contents in various mung bean genotypes. For instance, Wang et al. [29] found linolenic acid (38.95–44.74%) as the most abundant fatty acid across 24 mung bean genotypes. The same study reported comparable ranges of palmitic acid (24.81–27.33%), stearic acid (4.99–8.22%), oleic acid (2.69–8.17%), and linolenic acid (17.81–25.89%). Other studies also reported comparable contents of the five fatty acids to those obtained in this study [33,36,41]. This study also observed a wide variation in omega-6 (ω-6) to omega-3 (ω-3) fatty acids ratio, which ranged from 1.53 to 3.51 with a CV of 14.32% (Table 2 and Table S2). Plant-based fatty acids are becoming important, owing to their health benefits. Incorporating plant-based oils that have a lower ratio of ω-6 to ω-3 fatty acids in the diet has been proven to prevent various diseases, while oils with a higher ratio are linked to chronic illnesses. This highlights the importance of mung bean varieties with a lower ω-6 to ω-3 ratio as sources of nutrition and potential candidates for breeding [41,42]. On the other hand, legume oils that contain high levels of polyunsaturated fatty acids may have a reduced shelf life due to their susceptibility to oxidation [42]. As a result, those mung bean accessions with a lower double bond index could serve as valuable resources (Table S2).

### 3.4. Variations of Total Saponin and Total Phenol Contents

The TSC level ranged from 1.35 to 34.56 mg DE/g, while the TPC level ranged from 1.66 to 4.04 mg GAE/g, exhibiting over 25-fold and 2.5-fold variations, respectively (Table S1 and Table 3). Accession MB65 exhibited the highest TPC, while accession MB3 had the highest TSC. Conversely, accessions MB30 and MB10 had the lowest TSC and TPC, respectively. The contents of polyphenols and saponins in crops and foods are affected by differences in extraction processes and analysis protocols as well as genotype and environmental factors. These could cause discrepancies in the reported values, making it difficult to make reliable comparisons. Overall, the TPC and TSC ranges found in this study were comparable with many previous findings, although other studies also reported much higher and/or lower ranges [29,33,36,43,44]. Phenolic compounds and saponins found in several crops, in general, are known for their health benefits [8,9,10,17,29]. Interestingly, 27.94% of mung bean accessions had TPC and TSC levels above the average values (2.74 mg GAE/g and 7.37 mg DE/g, respectively). These accessions could be sources of beneficial phenolic compounds. In contrast, some specific phenolic compounds and saponins in legumes are considered anti-nutrients since they affect the availability and digestibility of nutrients [45]. In this regard, accessions with lower levels of these compounds could be valuable resources. In general, the findings of this study could serve as a basis to explore the specific metabolites with such undesirable properties and identify mung bean genotypes with lower levels of anti-nutrient factors.

### 3.5. Variations of Antioxidant Activities

The levels of DPPH^•^ scavenging activity, ABTS^•+^ scavenging activity, and FRAP ranged from 12.57 to 110.21 mg AAE/100 g, 81.96 to 446.38 mg TE/100 g, and 18.45 to 182.14 mg AAE/100 g, respectively, each showing more than 5-fold variation (Table S1 and Table 3). The discrepancies in the observed ranges among the different antioxidant assays could be attributed to their difference in the mechanism of action to scavenge reactive radicals [36,43,44]. Many previous studies also showed the antioxidant activities of mung bean genetic material and reported wide-ranging values due to discrepancies in the extraction protocols and reporting methods in addition to differences in genotypes and other environmental conditions [29,36,43,46]. Accession MB65 exhibited the highest DPPH^•^ scavenging activity, ABTS^•+^ scavenging activity, and FRAP values. It is important to note that this accession also exhibited the highest TPC level. Other accessions such as MB3, MB42, MB72, MB79, and MB80, which contained high levels of TPC and TSC, also exhibited strong antioxidant activities (Table S1). Interestingly, all of these accessions, except for MB72 and MB79, had vitexin and isovitexin contents greater than or equal to the average values. Conversely, accession MB22 showed the lowest DPPH^•^ scavenging activity and ABTS^•+^ scavenging activity, as well as the second lowest FRAP, with accession MB30 having the lowest FRAP. Once again, these accessions exhibited the lowest (MB30) and the second lowest (MB22) TSC in addition to their low TPC level. Accession MB30 also had vitexin and isovitexin levels much lower than the average values. Other accessions, including MB23, MB25, MB29, and MB34, which relatively contained low levels of TSC and/or TPC, also demonstrated low antioxidant activities (Table S1). Overall, these observations demonstrate the roles of such metabolites as antioxidants in mung beans despite their anti-nutrient effects [47,48]. Therefore, identifying specific metabolites responsible for such activities in the mung bean accessions could be a potential research focus in the future [2,8,33].

### 3.6. Effect of Seed Weight Difference on the Analyzed Parameters

Several studies revealed that seed-related traits, such as seed weight and seed color in legumes, are regulated by different types of genes and vary between genotypes [22,49,50]. These candidate genes were also found to influence the overall seed quality of legumes including metabolite contents. In relation to these, previous studies have investigated the effects of seed-related traits on the levels of different classes of metabolites in legumes [19,22,51,52,53]. In this study, the mung bean accessions showed a significant variation in their one-thousand seeds weight signifying genetic variance between them (Table S1). Accordingly, the mung bean accessions were classified as small, medium, and large as described before, and the effect of seed weight difference on the levels of nutritional components, flavonoids, total metabolite contents, and antioxidant activities was statistically analyzed [28]. Table 1 shows the variations of vitexin and isovitexin among mung beans of different seed weights. The results showed that seed weight difference had no significant effects on both vitexin and isovitexin levels. Previous studies have reported conflicting results regarding the effect of seed weight on the levels of specific metabolites in legumes and other crops [19,24,54]. For instance, Kim et al. [52] found significant variations in the levels of isoflavones in soybeans based on their seed weights. In a previous study, we also observed significant differences in the concentrations of isoflavones among black soybeans of different seed weights [55]. In contrast, Lee et al. [53] studied soybeans grown in different locations and found that seed weight difference had not a significant effect on the total isoflavone content. A recent study by He et al. [56] noted variations in vitexin and isovitexin levels in mung beans grown in different locations, despite the lack of statistical analysis to support the findings. The observed inconsistencies in the levels of individual metabolites in different legumes could be due to genetic variance, the year of cultivation, and treatment conditions that affect the stability of the compounds, among others [18,24,35]. In general, the results of this study suggest that vitexin and isovitexin levels in mung beans are not significantly affected by the difference in seed weight. As a result, seed weight may not be used as a reliable indicator to distinguish mung bean genetic materials based on their vitexin and isovitexin levels. These further highlight the importance of evaluating each mung bean variety separately.

The effect of seed weight difference on the nutritional components was also statistically analyzed (Table 2). While total protein content was not significantly affected by seed weight difference, total starch content showed a significant variation (*p* < 0.05). Accordingly, the average total starch content decreased in the order of large > small > medium seeds, the former being significantly different from the latter two. Previous studies on the effects of origin and seed weight on the levels of total protein in other legumes reported inconsistent findings [30,54,57,58]. Among the five fatty acids, palmitic acid, linoleic acid, and linolenic acid contents showed significant variations between small, medium, and large seeds (*p* < 0.05). In contrast, stearic acid and oleic acid remained unaffected by seed weight difference. Accordingly, small seeds had the highest average palmitic acid and linolenic acid contents, each being significantly different from those of medium and large seeds. Small seeds also displayed the lowest average linoleic acid content (*p* < 0.05). Medium seeds, on the other hand, exhibited the lowest average linolenic acid content, while large seeds had the lowest average palmitic acid content. In our previous study, the difference in seed weight significantly affected all individual soybean fatty acids except for stearic acid [30]. Moreover, Lee et al. [58] also found significant variations in all fatty acids except for linolenic acid among soybeans of different seed weights. Unlike the individual fatty acids, however, seed weight difference had no significant effect on the TUFA and TSFA (Table 2). Overall, the observed results signify that seed weight could be applicable for discriminating a large collection of mung beans based on the levels of total starch and individual fatty acids including palmitic acid, linoleic acid, and linolenic acid. The fact that seed weight difference showed a significant effect on the levels of linoleic acid and linolenic acid, the two essential fatty acids, signifies that it could also be used to estimate the ω-6:ω-3 ratio and, hence, the lipid quality of mung beans [38,42]. These observations could initiate future studies that focus on investigating the effects of seed weight and other seed-related factors on the levels of protein and starch components including individual amino acids and sugars [35,38,40].

The variations of total secondary metabolite contents and antioxidant activities among mung beans of different seed weights are summarized in Table 3. While TSC was significantly affected by seed weight difference, TPC remained unaffected (*p* < 0.05). Specifically, the average TSC decreased in the order of small > medium > large seeds. Previous studies also showed that small legumes, such as soybeans, contain high levels of phenolic compounds due to their large surface area-to-volume ratio [22,53]. Statistical analysis showed that seed weight difference had also a significant effect on DPPH^•^ scavenging activity and FRAP. In contrast, ABTS^•+^ scavenging activity was not significantly affected. Specifically, small seeds showed the highest average DPPH^•^ scavenging activity and FRAP. Large seeds had the lowest average values of all the antioxidant activities (Table 3). Studies on the effects of seed weight on the levels of antioxidant activities in mung beans and other legumes are scarce. Our previous studies on different soybean genotypes found that seed weight difference had no significant effect on any of the antioxidant activities [30,55]. Overall, the results of this study suggest that seed weight could be used as a distinguishing factor for classifying mung bean genetic materials based on their total saponin level, DPPH^•^ scavenging activity, and FRAP. Moreover, the results of this study highlighted the variable effect of seed weight on the antioxidant activities of mung beans.

### 3.7. Multivariate Analysis

**Figure 1:**
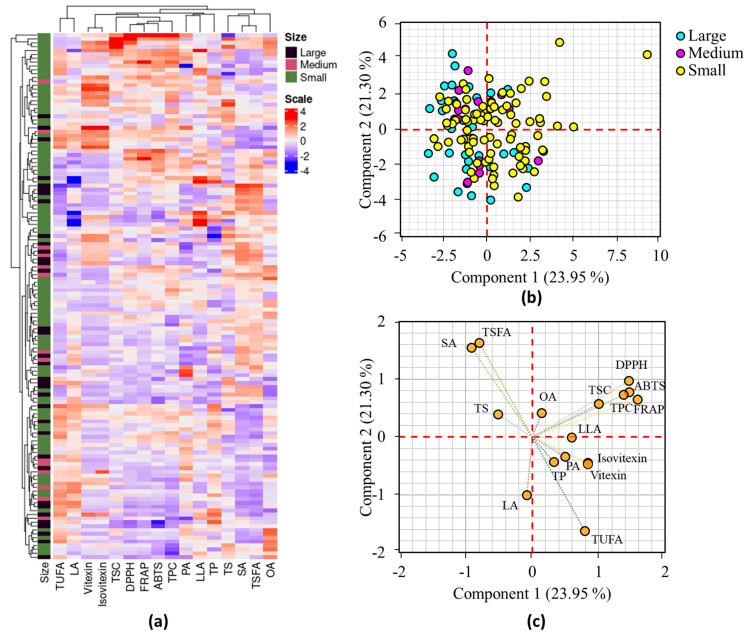
Hierarchical cluster analysis matrix (**a**), score plot of mung bean accessions based on seed weight (**b**), and loading plot of variables (**c**) obtained from PCA. ABTS: ABTS^•+^ scavenging activity, DPPH: DPPH^•^ scavenging activity, LA: linoleic acid, LLA: linolenic acid, OA: oleic acid, PA: palmitic acid, FRAP: ferric reducing antioxidant power, SA: stearic acid, TP: total protein, TPC: total phenolic content, TS: total starch, TSC: total saponin content, TSFA: total saturated fatty acid, TUFA: total unsaturated fatty acid.

Multivariate statistical analysis methods such as HCA, PCA, and correlation analysis were applied to investigate the distribution of the mung bean accessions and their associations with all analyzed parameters [15,18]. Figure 1a shows the HCA result and the mung bean accessions were not clearly grouped according to their seed weight.

**Figure 2:**
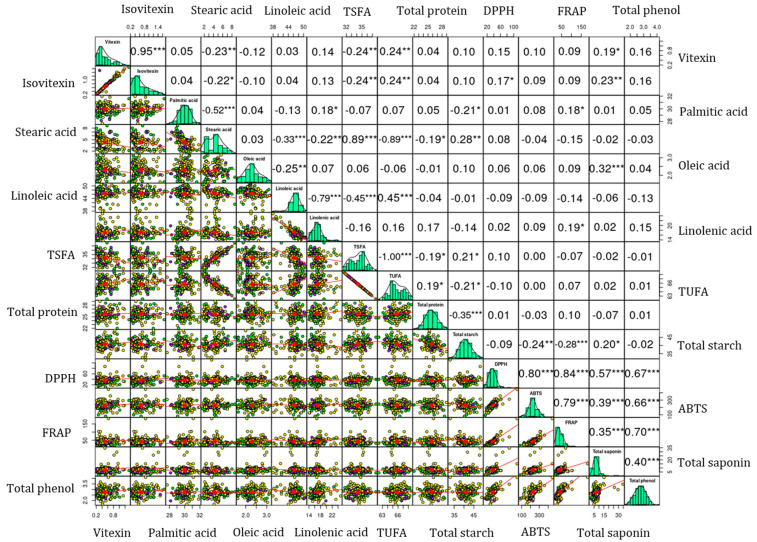
Pearson’s correlation matrix showing the association between the analyzed biochemical parameters. Small mung beans: Yellow, Medium mung beans: Purple; Large mung beans: Green. *** *p* < 0.001, ** *p* < 0.01, * *p* < 0.05. ABTS: ABTS^•+^ scavenging activity, DPPH: DPPH^•^ scavenging activity, FRAP: ferric reducing antioxidant power, TSFA: total saturated fatty acid, and TUFA: total unsaturated fatty acid.

Despite this, there was a clear grouping of small mung beans into two separate clusters. Additionally, the HCA grouped variables such as TPC, TSC, DPPH^•^ scavenging activity, ABTS^•+^ scavenging activity, and FRAP, while vitexin and isovitexin were categorized in a separate group. OA was clearly distinguished from the other fatty acids in the HCA (Figure 1a). The PCA findings were also in agreement with the HCA results. The PCA identified six components with Eigen values above 1, which accounted for 83.60% of the overall variance. The first two components (PC1 and PC2), in particular, explained 45.25% of the total variance and were used for further analysis. As shown in the score plot (Figure 1b), the mung bean accessions were widely dispersed along the axis of PC1 and PC2. Despite this, most of the large and medium-sized mung beans were clustered on the negative side of PC1. The main factors that had FL values exceeding ±0.50 and high contribution to the variance observed in PC1 were TPC, TSC, ABTS^•+^ scavenging activity, and FRAP, accounting for 7.11 to 17.82%. Conversely, stearic acid, linoleic acid, TSFA, and TUFA were the primary contributors to the variance observed along PC2, with contributions ranging from 8.05 to 21.10% (Table S3). DPPH^•^ scavenging activity displayed FL values greater than ±0.50 in both PC1 and PC2, contributing 14.99% and 7.51%, respectively. The grouping of the parameters examined in the loading plot was also comparable to the HCA observation (Figure 1c). The degree of correlation among the parameters examined was assessed using Pearson’s correlation analysis (Figure 2).

Vitexin and isovitexin exhibited a significant and positive correlation with each other (r = 0.95, *p* < 0.001). Similar correlation values were observed irrespective of seed weight difference (Figure S2). Both vitexin and isovitexin were closely clustered in the HCA and the loading plot of the PCA. These observations could signify their interrelated biosynthesis pathways. Both vitexin and isovitexin exhibited positive correlations with DPPH^•^ scavenging activity, the latter showing a significant correlation (*p* < 0.05). In contrast, the correlations of both compounds with ABTS^•+^ scavenging activity and FRAP were not significant. These observations could indicate the different mechanisms of the action of vitexin and isovitexin against reactive radicals [13,33,44]. TPC and TSC also exhibited moderate to strong correlations (0.35 ≤ r ≤ 0.65) with each other in the whole population and among mung beans with varying seed weights. Additionally, these metabolites demonstrated strong correlations with each of the antioxidant activities (0.35 ≤ r ≤ 0.88) at different significance levels. These results support the outcomes of the HCA and PCA (Figure 1c). Furthermore, such strong and significant associations suggest that these metabolites have a role in combating reactive radicals as observed in prior studies and, hence, could lead to further metabolomics studies [29,36,46,48]. Among the fatty acids, oleic acid demonstrated a weak or negative relationship with the other individual fatty acids. In addition, linoleic acid displayed a negative correlation with linolenic acid (r = −0.79, *p* < 0.001). These could be attributed to the actions of fatty acid desaturase enzymes that regulate the interconversion of unsaturated fatty acids in legumes [41,42]. Overall, flavonoids, nutritional components, total secondary metabolites, and antioxidant activities showed different levels of correlations supporting the HCA and PCA observations.

## 4. Conclusions

Different factors related to environment and genetics affect the distributions and contents of health-promoting secondary metabolites and nutritional components in crops. With these, several studies conducted on different types of legumes have demonstrated the effects of seed-related traits, origin, growing conditions, and post-harvest handling processes. This study investigated the variations of major flavonoids, nutritional components, total secondary metabolite contents, and antioxidant activities in 136 mung bean accessions and statistically investigated the effect of seed weight difference on each. All the analyzed parameters including the contents vitexin, isovitexin, total protein, total starch, fatty acids (palmitic acid, stearic acid, oleic acid, linoleic acid, and linolenic acid), total phenol, total saponin, and antioxidant activities (DPPH^•^ scavenging activity, ABTS^•+^ scavenging activity, and ferric reducing antioxidant power) showed significant variations indicating genetic variances among the mung bean accessions. Furthermore, statistical analysis exhibited that seed weight difference had a significant effect on total starch content, all individual fatty acids except for stearic acid and oleic acid, total saponin content, and all antioxidant activities except for ABTS^•+^ scavenging activity. In contrast, the other parameters including vitexin, isovitexin, total protein, total phenol, and total fatty acid contents remained unaffected by seed weight difference. In general, the results of this study showed the variable effects of seed weight difference on major flavonoids, nutritional components, total secondary metabolite contents, and antioxidant activities in mung bean genetic materials. The observed results could serve as a basis for future metabolomics and genomics studies in mung bean genotypes.

## Figures and Tables

**Table 1:** Variations of vitexin and isovitexin concentrations in small, medium, and large mung bean seeds.

Seed Weight	Vitexin (mg/g dw)				CV	Isovitexin (mg/g dw)				CV
	Min.	Max.	Mean	SD		Min.	Max.	Mean	SD	
Small	0.22	1.18	0.52 ^a^	0.23	45.44	0.26	1.58	0.67 ^a^	0.26	49.89
Medium	0.22	0.95	0.47 ^a^	0.22	46.39	0.29	1.50	0.68 ^a^	0.29	53.09
Large	0.21	1.33	0.48 ^a^	0.27	56.04	0.27	1.67	0.61 ^a^	0.27	56.37
Total	0.21	1.33	0.50	0.21	48.41	0.26	1.67	0.66	0.34	51.95

Different superscript letters in a column represent significantly different means (*p* < 0.05). CV: coefficient of variation, SD: standard deviation.

**Table 2:** Variations of total protein, total starch, and fatty acid contents in small, medium, and large mung bean seeds.

Parameter	Values	Seed Weight			Total
		Small	Medium	Large	
Total protein (g/100 g)	Min.	22.71	24.73	22.01	22.01
	Max.	28.96	27.58	28.55	28.96
	Mean	25.73 ^a^	25.90 ^a^	25.51 ^a^	25.69
	SD	1.23	0.83	1.37	1.24
	CV	4.78	3.21	5.37	4.82
Total starch (g/100 g)	Min.	32.62	36.84	39.45	32.62
	Max.	49.03	44.43	46.93	49.03
	Mean	40.41 ^b^	40.17 ^b^	42.22 ^a^	40.85
	SD	3.27	2.02	2.06	3.00
	CV	8.09	5.03	4.87	7.34
Palmitic acid (g/100 g)	Min.	27.71	28.16	28.45	27.71
	Max.	31.94	30.57	31.59	31.94
	Mean	30.13 ^a^	29.67 ^b^	29.67 ^b^	29.96
	SD	0.67	0.73	0.77	0.74
	CV	2.22	2.48	2.60	2.46
Stearic acid (g/100 g)	Min.	1.84	2.43	1.62	1.62
	Max.	7.01	7.12	8.61	8.61
	Mean	4.05 ^a^	4.72 ^a^	4.64 ^a^	4.27
	SD	1.26	1.68	2.08	1.59
	CV	31.20	35.65	44.91	37.13
Oleic acid (g/100 g)	Min.	1.62	1.76	1.62	1.62
	Max.	3.15	2.93	3.10	3.15
	Mean	2.36 ^a^	2.19 ^a^	2.21 ^a^	2.30
	SD	0.35	0.30	0.37	0.36
	CV	14.65	13.56	16.79	15.46
Linoleic acid (g/100 g)	Min.	37.96	44.76	39.52	37.96
	Max.	50.67	50.21	50.71	50.71
	Mean	45.92 ^b^	47.26 ^a^	47.11 ^a^	46.36
	SD	2.39	1.49	2.10	2.32
	CV	5.20	3.15	4.46	5.00
Linolenic acid (g/100 g)	Min.	13.78	14.24	14.12	13.78
	Max.	24.89	19.83	22.55	24.89
	Mean	17.54 ^a^	16.16 ^b^	16.38 ^b^	17.10
	SD	2.08	1.33	1.81	2.04
	CV	11.87	8.22	11.03	11.91
TSFA (g/100 g)	Min.	31.47	31.73	32.46	31.47
	Max.	37.68	36.56	36.63	37.68
	Mean	34.23 ^a^	34.18 ^a^	34.39 ^a^	34.30
	SD	1.35	1.18	1.37	1.69
	CV	3.95	3.46	4.00	4.00
TUFA (g/100 g)	Min.	62.32	63.44	63.37	62.32
	Max.	68.53	68.27	67.54	68.53
	Mean	65.77 ^a^	65.82 ^a^	65.61 ^a^	65.77
	SD	1.35	1.18	1.37	1.35
	CV	2.06	1.80	2.10	2.57
ω-6:ω-3 ratio	Min.	1.53	2.26	1.75	1.53
	Max.	3.51	3.43	3.41	3.51
	Mean	2.66 ^b^	2.95 ^a^	2.92 ^a^	2.76
	SD	0.39	0.27	0.37	0.39
	CV	14.65	9.31	12.61	14.32

Different superscript letters in a row represent significantly different means (*p* < 0.05). CV: coefficient of variation, TSFA: total saturated fatty acid, TUFA: total unsaturated fatty acid, SD: standard deviation.

**Table 3:** Variations of total saponin content, total phenolic content, and antioxidant activities in small, medium, and large mung bean seeds.

Parameter	Values	Seed Weight			Total
		Small	Medium	Large	
TSC (mg DE/g)	Min.	1.52	2.73	1.35	1.35
	Max.	34.56	9.51	9.47	34.56
	Mean	8.06 ^a^	6.43 ^ab^	6.02 ^b^	7.37
	SD	4.95	2.41	1.86	4.25
	CV	61.41	37.54	30.97	57.64
TPC (mg GAE/100 g)	Min.	1.66	1.96	2.14	1.66
	Max.	4.09	2.97	3.28	4.09
	Mean	2.80 ^a^	2.60 ^a^	2.64 ^a^	2.74
	SD	0.45	0.28	0.32	0.41
	CV	16.13	10.93	12.04	15.12
DPPH (mg AAE/100 g)	Min.	18.15	26.25	12.57	12.57
	Max.	110.21	49.49	52.63	110.21
	Mean	38.44 ^a^	37.27 ^ab^	32.01 ^b^	36.66
	SD	13.67	7.36	9.67	12.52
	CV	35.56	19.74	30.22	34.15
ABTS (mg TE/100 g)	Min.	124.07	196.12	81.96	81.96
	Max.	446.38	306.49	296.02	446.38
	Mean	233.55 ^a^	234.14 ^a^	211.46 ^a^	227.93
	SD	54.10	33.47	45.91	51.23
	CV	23.16	14.29	21.71	22.47
FRAP (mg AAE/100 g)	Min.	23.23	24.18	18.45	18.45
	Max.	182.14	67.84	58.68	182.14
	Mean	47.47 ^a^	39.21 ^ab^	33.08 ^b^	42.92
	SD	21.99	12.75	11.42	19.98
	CV	46.32	32.52	34.51	46.55

Different superscript letters in a row represent significantly different means (*p* < 0.05). ABTS: ABTS^•+^ scavenging activity, CV: coefficient of variation, DPPH: DPPH^•^ scavenging activity, FRAP: ferric reducing antioxidant power, TPC: total phenolic content, TSC: total saponin content.

## Data Availability

All the data related to this study are incorporated in the manuscript and Supplementary Materials. Further inquiries can be directed to the corresponding authors.

## Supplementary Materials

The following supporting information can be downloaded at https://www.mdpi.com/article/10.3390/foods13213387/s1. Figure S1. HPLC–DAD chromatograms of vitexin and isovitexin standards mixture (a) and representative small (b), medium (c), and large (d) mung bean seed samples. Figure S2. Pearson’s correlation matrix in small (a), medium (b), and large (c) mung beans. Table S1. General information of 136 mung bean accessions and their flavonoid contents, total secondary metabolite contents, and antioxidant activities. Table S2. Nutritional and fatty acid contents of 136 mung bean accessions. Table S3. Factor loadings and contributions of variables in the first six principal components.
