# Supplementary material for: Variations of Major Flavonoids, Nutritional Components, and Antioxidant Activities in Mung Beans (Vigna radiate L.) of Different Seed Weights
Source: Foods. 2024 Oct 24;13(21):3387. doi: 10.3390/foods13213387 (PMC11545297; doi:10.3390/foods13213387)
Supplement: Supplementary file 1 [file foods-13-03387-s001.zip › foods-3265453-supplementary.pdf]

Supplementary material

Variations of Major Flavonoids, Nutritional Components, and Antioxidant Activities in Mung Beans (*Vigna radiate* L.) of Different Seed Weights

Kebede Taye Desta [1,2,†], Yu-Mi Choi [1,†], Jungyoon Yi [1], Myoung-Jae Shin [1], Young-ah Jeon [1] and Hyemyeong Yoon [1,*]

1 National Agrobiodiversity Center, National Institute of Agricultural Sciences, Rural Development Administration, Jeonju 54874, Republic of Korea; kebedetdesta@korea.kr (K.T.D.); cym0421@korea.kr (Y.-M.C.); naaeskr@korea.kr (J.Y.); smj1204@korea.kr (M.-J.S.); yjeon@korea.kr (Y.-a.J.)
2 Department of Applied Chemistry, College of Natural and Computational Sciences, Adama Science and Technology University, Adama P.O. Box 1888, Ethiopia
* Correspondence: mmihm@korea.kr
† These authors contributed equally to this work.

Abbreviations used in the supplementary material

ABTS: $ABTS^{\bullet+}$ scavenging activity
CV: Coefficient of variation
DBI: Double bond index
DPPH: $DPPH^{\bullet}$ scavenging activity
FRAP: Ferric reducing antioxidant power
LA: Linoleic acid
LLA: Linolenic acid
OA: Oleic acid
PA: Palmitic acid
SA: Stearic acid
SD: Standard deviation
TP: Total protein
TPC: Total phenol
TS: Total starch
TSC: Total saponin
TSFA: Total saturated fatty acid
TSW: One-thousand seeds weight.
TUFA: Total unsaturated fatty acid

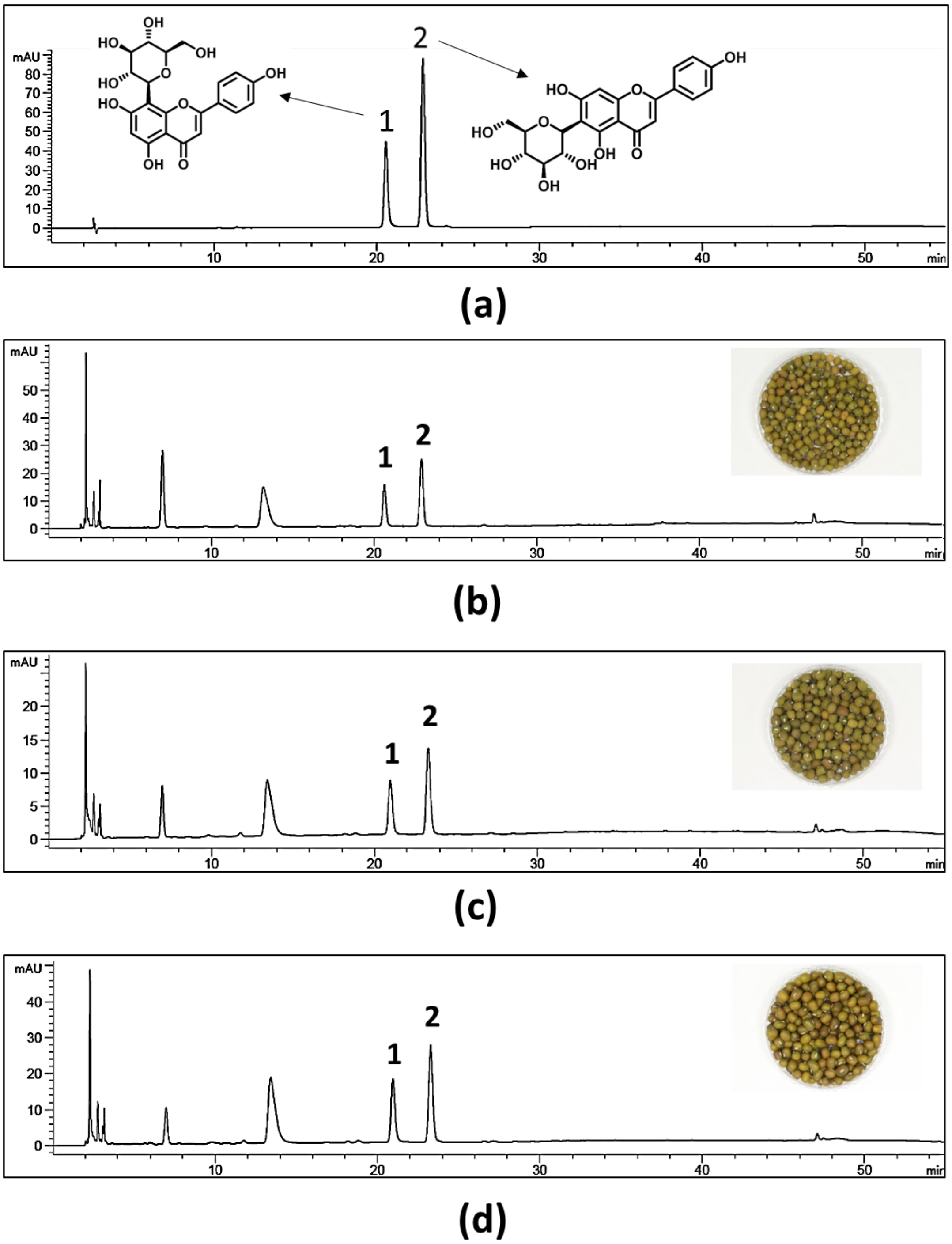

Figure S1. HPLC-DAD chromatograms of vitexin and isovitexin standards mixture (a) and representative small (b), medium (c), and large (d) mung bean seed samples. Peak assignment: Vitexin (1), Isovitexin (2).

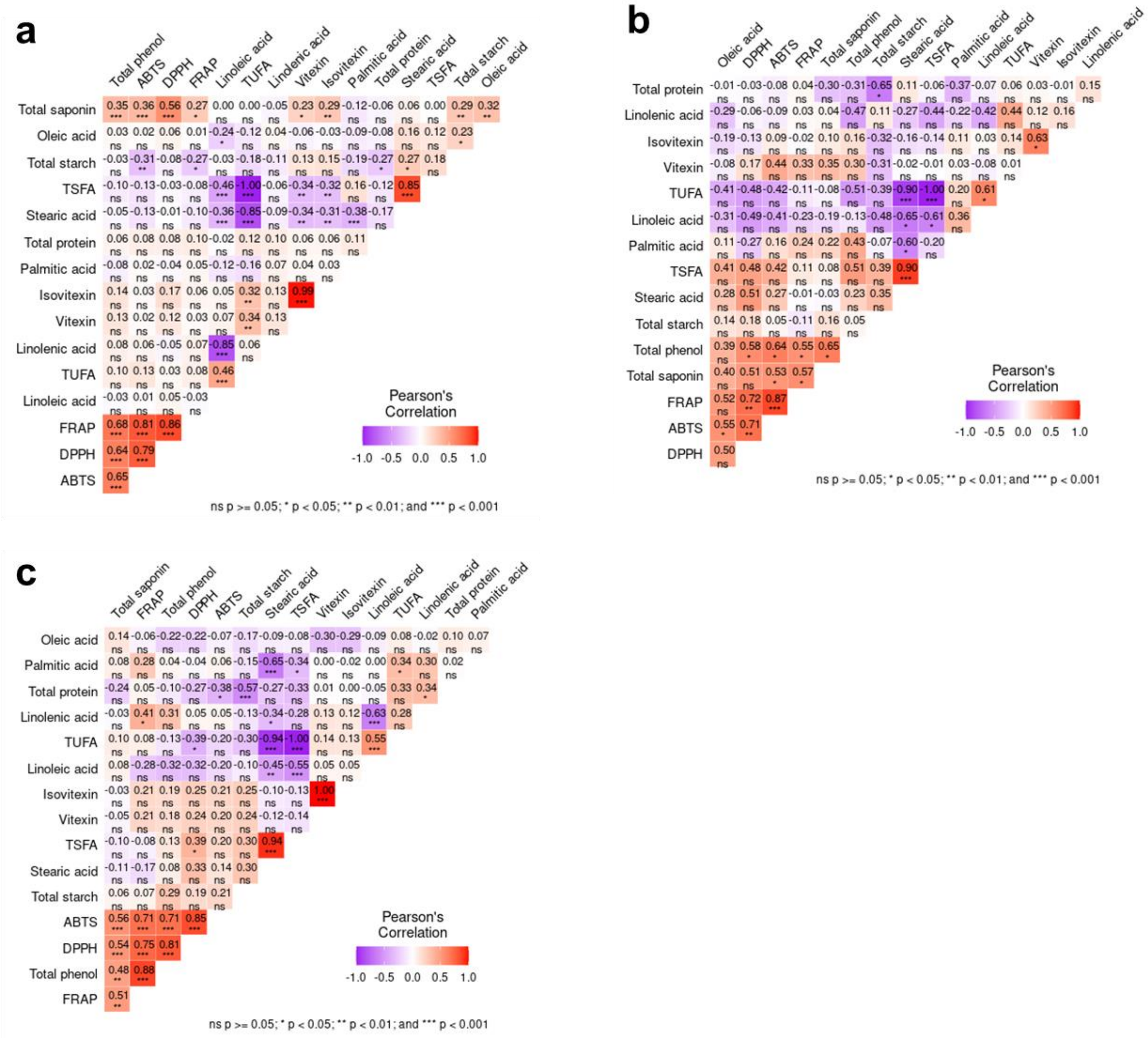

Figure S2. Pearson's correlation matrix in small (a), medium (b), and large (c) mung beans.

Table S1. General information on 136 mung bean accessions and their flavonoid contents, total secondary metabolite contents, and antioxidant activities.

General information					Flavonoid contents (mg/g dw)		Total metabolite contents		Antioxidant activities		
Name	IT number	Code	TSW (g)	Size	Vitexin	Isovitexin	TSC (mg TE/g)	TPC (mg GAE/100g)	DPPH (mg AAE/100g)	ABTS (mg TE/100g)	FRAP (mg AAE/100g)
Chungbukjaerae-9ho	26025	MB1	43.00 ± 1.41	Small	0.82 ± 0.06	1.20 ± 0.09	25.83 ± 6.62	318.46 ± 7.46	46.92 ± 4.79	197.75 ± 8.44	33.83 ± 2.50
Chungbukjaerae-12ho	26028	MB2	41.33 ± 0.47	Small	0.91 ± 0.11	1.42 ± 0.18	29.55 ± 5.74	297.98 ± 46.33	67.07 ± 20.06	246.64 ± 76.30	51.72 ± 15.57
Gyeonggijaerae-13ho	26058	MB3	36.00 ± 1.41	Small	0.50 ± 0.07	0.72 ± 0.10	34.56 ± 0.45	347.17 ± 3.56	86.77 ± 6.32	312.42 ± 12.52	70.58 ± 6.62
PI377228	26202	MB4	46.67 ± 0.47	Small	0.35 ± 0.03	0.48 ± 0.04	9.00 ± 1.19	209.07 ± 8.42	42.85 ± 8.56	228.84 ± 35.12	47.65 ± 8.23
PI377235	26203	MB5	35.33 ± 0.47	Small	0.54 ± 0.04	0.79 ± 0.06	8.63 ± 1.06	195.91 ± 29.19	34.28 ± 14.17	178.52 ± 48.82	40.80 ± 15.75
EC27087-2	26218	MB6	59.67 ± 1.89	Medium	0.37 ± 0.01	0.51 ± 0.03	3.87 ± 0.42	195.97 ± 3.37	41.35 ± 0.57	226.05 ± 9.14	42.14 ± 0.57
EC27261-3	26219	MB7	41.33 ± 5.79	Small	0.85 ± 0.09	1.21 ± 0.10	7.41 ± 0.80	227.44 ± 14.64	41.15 ± 14.08	228.79 ± 52.89	45.88 ± 13.75
V1842	26223	MB8	46.67 ± 1.25	Small	0.35 ± 0.01	0.47 ± 0.02	6.98 ± 1.16	213.41 ± 9.19	48.97 ± 10.79	232.92 ± 35.97	49.95 ± 13.23
V2273	26228	MB9	40.67 ± 0.47	Small	0.43 ± 0.02	0.56 ± 0.03	5.79 ± 1.14	183.43 ± 21.04	31.69 ± 9.74	167.06 ± 43.23	32.72 ± 10.22
V5197	26233	MB10	39.33 ± 1.70	Small	0.42 ± 0.05	0.55 ± 0.07	4.77 ± 1.34	165.99 ± 7.53	29.54 ± 2.15	155.94 ± 14.79	27.15 ± 0.53
Jeonbukiksan-1985-1085	101085	MB11	49.00 ± 1.63	Small	0.35 ± 0.01	0.46 ± 0.01	7.11 ± 0.36	191.17 ± 13.07	34.56 ± 6.12	186.66 ± 25.82	34.75 ± 6.70
Jeonbukiksan-1985-2597	102597	MB12	39.33 ± 0.47	Small	0.48 ± 0.05	0.67 ± 0.08	11.19 ± 0.83	325.37 ± 28.55	49.69 ± 16.98	232.31 ± 41.58	58.74 ± 16.38
Gyeonggianseong-1985-2751	102751	MB13	43.33 ± 0.47	Small	0.37 ± 0.06	0.50 ± 0.08	10.58 ± 0.12	330.49 ± 36.40	41.08 ± 13.77	235.72 ± 45.98	58.62 ± 15.73
Gyeongbukyecheon-1985-2800	102800	MB14	51.00 ± 0.82	Small	0.42 ± 0.03	0.56 ± 0.04	4.85 ± 0.92	278.09 ± 41.17	28.07 ± 8.90	185.75 ± 43.60	44.07 ± 14.86
Gyeongnamchangneong-1985-2855	102855	MB15	68.67 ± 0.47	Large	0.70 ± 0.05	0.90 ± 0.06	6.76 ± 0.91	280.50 ± 23.27	30.28 ± 12.69	186.00 ± 40.59	39.52 ± 13.54
Gyeongbukdalseong-1985-4003	104003	MB16	37.33 ± 0.94	Small	0.92 ± 0.09	1.18 ± 0.15	8.69 ± 0.43	239.68 ± 0.87	19.60 ± 0.52	126.47 ± 0.53	35.70 ± 0.09
Gyeonggipyeongtaek-1985-4148	104183	MB17	40.67 ± 0.47	Small	0.46 ± 0.04	0.61 ± 0.06	10.31 ± 0.30	308.31 ± 1.37	20.52 ± 1.87	133.87 ± 4.01	33.69 ± 2.16
Jeonbukwanju-1985-9145	109145	MB18	62.00 ± 2.45	Large	0.35 ± 0.04	0.44 ± 0.05	6.95 ± 0.59	323.44 ± 26.29	45.16 ± 10.62	237.50 ± 38.73	58.68 ± 14.07
Goribnokdu	111041	MB19	39.67 ± 0.47	Small	0.42 ± 0.01	0.55 ± 0.02	6.58 ± 1.65	313.83 ± 35.16	42.01 ± 9.93	239.04 ± 44.59	49.77 ± 15.90
Gyeonggihwangseong-1985-12822	112822	MB20	37.33 ± 0.47	Small	0.33 ± 0.01	0.42 ± 0.01	5.76 ± 1.23	290.31 ± 29.89	41.67 ± 11.52	223.57 ± 42.75	57.59 ± 15.04
Gangwonhongcheon-1985-12930	112930	MB21	45.67 ± 0.47	Small	0.39 ± 0.04	0.53 ± 0.06	5.38 ± 1.02	307.47 ± 11.60	28.89 ± 4.31	179.44 ± 20.63	38.18 ± 5.12
Gyeongbukseongju-1986-24373	138114	MB22	62.00 ± 0.82	Large	0.53 ± 0.03	0.66 ± 0.04	1.44 ± 0.18	218.88 ± 6.24	12.57 ± 0.89	81.96 ± 1.52	19.17 ± 1.42
V2272	145272	MB23	35.67 ± 0.47	Small	0.42 ± 0.02	0.49 ± 0.04	4.30 ± 0.99	223.53 ± 2.85	24.29 ± 0.52	124.07 ± 1.40	28.08 ± 0.42
V3726	145275	MB24	71.67 ± 1.25	Large	0.57 ± 0.04	0.71 ± 0.05	4.79 ± 0.70	289.14 ± 13.53	25.16 ± 2.61	137.27 ± 9.41	32.47 ± 2.63
V2772	145310	MB25	35.33 ± 0.47	Small	0.31 ± 0.03	0.39 ± 0.04	1.52 ± 0.14	263.96 ± 10.58	23.38 ± 0.33	126.82 ± 7.60	28.80 ± 1.73
V4668	145311	MB26	41.33 ± 2.62	Small	0.85 ± 0.08	1.13 ± 0.12	4.71 ± 0.45	350.30 ± 33.50	48.56 ± 11.74	217.80 ± 35.61	56.80 ± 13.25

V5000	145312	MB27	43.67 ± 0.47	Small	0.98 ± 0.07	1.36 ± 0.09	4.48 ± 0.92	337.31 ± 18.58	40.89 ± 6.59	216.08 ± 16.78	49.12 ± 6.40
KLM87057	154071	MB28	50.00 ± 0.82	Small	0.58 ± 0.08	0.74 ± 0.12	4.11 ± 0.53	322.07 ± 35.41	36.43 ± 12.09	186.12 ± 42.24	46.00 ± 12.61
Jeongbukjeongup-1989-5463	162743	MB29	36.00 ± 0.82	Small	0.78 ± 0.09	1.05 ± 0.13	1.99 ± 0.38	258.11 ± 6.40	18.15 ± 1.17	125.53 ± 6.43	23.23 ± 1.39
Vo1301	163175	MB30	61.67 ± 0.47	Large	0.28 ± 0.02	0.33 ± 0.02	1.35 ± 0.28	222.48 ± 2.03	14.82 ± 1.26	99.62 ± 1.34	18.45 ± 0.39
Vo1869	163184	MB31	32.33 ± 0.47	Small	0.51 ± 0.07	0.63 ± 0.08	4.99 ± 0.69	271.40 ± 6.43	26.74 ± 1.82	173.47 ± 7.17	34.03 ± 2.47
Vo2273	163196	MB32	41.00 ± 0.00	Small	0.29 ± 0.00	0.34 ± 0.00	4.87 ± 0.89	339.81 ± 45.63	46.19 ± 16.59	223.81 ± 59.65	47.78 ± 15.28
Vo2366	163199	MB33	30.67 ± 0.94	Small	0.33 ± 0.02	0.37 ± 0.03	3.47 ± 1.40	269.45 ± 35.30	35.18 ± 11.54	186.96 ± 38.88	37.37 ± 12.07
Vo2850	163211	MB34	40.00 ± 0.82	Small	0.69 ± 0.02	0.88 ± 0.03	3.00 ± 0.53	209.02 ± 15.18	18.98 ± 3.53	165.08 ± 18.96	23.65 ± 5.92
Vo2866	163212	MB35	35.33 ± 1.25	Small	0.68 ± 0.05	0.85 ± 0.06	4.57 ± 0.46	211.39 ± 1.54	24.18 ± 0.74	197.68 ± 3.95	32.32 ± 1.10
Vo3406	163230	MB36	57.33 ± 1.25	Medium	0.36 ± 0.04	0.44 ± 0.05	8.10 ± 3.05	285.36 ± 49.29	45.64 ± 18.08	306.49 ± 74.89	67.84 ± 29.60
Vo3417	163232	MB37	31.00 ± 1.41	Small	0.29 ± 0.03	0.35 ± 0.04	6.71 ± 0.97	300.99 ± 58.84	61.91 ± 21.34	312.48 ± 65.55	103.90 ± 41.45
Vo4063	163247	MB38	36.00 ± 0.00	Small	0.25 ± 0.03	0.29 ± 0.03	5.71 ± 1.14	283.26 ± 46.69	39.63 ± 11.47	274.61 ± 60.08	53.04 ± 18.42
Vo4141	163251	MB39	45.00 ± 0.00	Small	0.22 ± 0.01	0.26 ± 0.01	4.97 ± 0.25	305.60 ± 8.34	44.67 ± 2.93	293.72 ± 15.61	59.17 ± 6.95
Vo4579	163259	MB40	32.33 ± 1.25	Small	0.32 ± 0.01	0.37 ± 0.01	8.27 ± 1.86	283.21 ± 9.39	32.87 ± 2.27	262.42 ± 8.60	46.72 ± 5.18
Vo4631	163261	MB41	33.00 ± 0.82	Small	0.31 ± 0.02	0.38 ± 0.02	5.69 ± 0.50	312.23 ± 7.45	49.68 ± 8.27	312.96 ± 17.40	84.33 ± 13.10
Vo4966	163270	MB42	36.67 ± 0.47	Small	0.69 ± 0.02	0.86 ± 0.04	7.00 ± 0.75	335.72 ± 8.64	59.28 ± 3.16	356.79 ± 10.66	81.03 ± 6.82
Vo4967	163271	MB43	46.00 ± 0.82	Small	0.28 ± 0.02	0.34 ± 0.03	5.50 ± 2.15	292.61 ± 34.51	46.20 ± 14.39	301.54 ± 69.22	60.51 ± 22.50
Vo1893	163292	MB44	42.00 ± 0.82	Small	0.29 ± 0.04	0.39 ± 0.06	6.77 ± 0.91	238.61 ± 18.93	22.94 ± 3.36	213.84 ± 15.25	27.35 ± 4.12
Vo1918	163294	MB45	29.67 ± 0.94	Small	0.54 ± 0.09	0.37 ± 0.05	10.32 ± 1.95	302.53 ± 34.68	35.47 ± 11.78	245.54 ± 48.56	45.78 ± 19.68
Vo1922	163295	MB46	38.33 ± 1.25	Small	0.40 ± 0.05	0.49 ± 0.07	8.53 ± 0.56	303.36 ± 22.71	31.91 ± 7.19	229.50 ± 34.51	41.41 ± 11.89
Vo2043	163308	MB47	41.00 ± 0.00	Small	0.35 ± 0.05	0.42 ± 0.05	9.58 ± 1.64	276.45 ± 21.83	36.74 ± 7.40	257.86 ± 39.96	48.54 ± 12.16
Vo2082	163309	MB48	43.33 ± 1.89	Small	0.57 ± 0.05	0.72 ± 0.07	9.13 ± 1.77	306.53 ± 52.03	36.02 ± 12.18	267.16 ± 56.23	47.93 ± 18.25
V4668	163331	MB49	39.33 ± 0.47	Small	0.64 ± 0.07	0.85 ± 0.08	10.36 ± 2.06	264.91 ± 41.26	29.72 ± 9.34	242.26 ± 31.83	34.21 ± 13.56
V2272	163336	MB50	40.67 ± 1.25	Small	0.54 ± 0.04	0.68 ± 0.05	11.39 ± 1.36	298.75 ± 23.76	35.60 ± 7.36	279.81 ± 31.53	46.25 ± 11.14
V2773	163339	MB51	65.00 ± 0.82	Large	0.27 ± 0.02	0.31 ± 0.02	7.48 ± 2.68	295.24 ± 55.20	41.93 ± 17.94	275.67 ± 68.46	58.16 ± 26.97
V3726	163344	MB52	44.67 ± 0.47	Small	0.25 ± 0.02	0.32 ± 0.03	6.56 ± 0.66	287.43 ± 37.39	30.46 ± 8.56	231.02 ± 44.20	41.14 ± 14.92
MBLS90-19	168064	MB53	62.00 ± 0.82	Large	0.26 ± 0.04	0.31 ± 0.04	5.97 ± 1.16	213.76 ± 19.31	17.34 ± 0.85	193.30 ± 4.01	19.21 ± 1.16
MBLS90-30	168075	MB54	66.67 ± 3.40	Large	0.24 ± 0.04	0.27 ± 0.04	5.68 ± 0.17	278.82 ± 8.37	30.94 ± 2.01	234.75 ± 7.93	35.55 ± 2.97
Gyeongbukcheongsong-1992-2658	175816	MB55	40.00 ± 0.00	Small	0.22 ± 0.00	0.27 ± 0.01	6.04 ± 0.58	235.14 ± 7.15	23.62 ± 0.59	204.79 ± 8.25	30.17 ± 3.00
Chungnamgeumsan-1993-43	178403	MB56	23.67 ± 0.47	Small	0.33 ± 0.05	0.43 ± 0.07	8.40 ± 0.96	288.00 ± 13.44	41.50 ± 3.23	267.54 ± 11.08	62.40 ± 6.11

Nongkwawon-1993-302	178662	MB57	50.33 ± 0.47	Small	0.53 ± 0.05	0.65 ± 0.09	8.51 ± 1.96	278.30 ± 47.77	39.56 ± 13.41	290.50 ± 67.48	54.79 ± 22.17
Gyeongbuksangju-1993-2473	180833	MB58	55.33 ± 0.94	Medium	0.78 ± 0.13	0.97 ± 0.16	9.12 ± 0.31	295.77 ± 27.52	41.39 ± 5.77	278.98 ± 25.46	57.28 ± 11.31
Gyeongbukgeumreung-1993-2475	180835	MB59	47.67 ± 1.25	Small	0.45 ± 0.05	0.54 ± 0.06	8.11 ± 2.53	286.46 ± 46.48	42.33 ± 15.30	316.21 ± 63.21	60.65 ± 26.54
Kyomo-5	183235	MB60	51.67 ± 0.94	Small	0.31 ± 0.02	0.38 ± 0.03	7.70 ± 0.43	247.92 ± 3.08	28.43 ± 0.08	224.09 ± 2.27	34.48 ± 1.69
F-Yonginkun	183238	MB61	49.00 ± 0.82	Small	0.42 ± 0.04	0.50 ± 0.05	4.45 ± 0.68	240.76 ± 12.55	26.45 ± 2.23	230.08 ± 13.88	30.10 ± 2.31
Gyeongnamchangneong-1994-3224	185563	MB62	57.67 ± 0.47	Medium	0.31 ± 0.04	0.39 ± 0.04	5.62 ± 1.36	254.63 ± 8.48	28.26 ± 1.67	231.30 ± 12.36	30.84 ± 3.47
Jeonnamdamyang-1994-3231	185570	MB63	24.00 ± 0.82	Small	0.90 ± 0.05	1.31 ± 0.08	5.70 ± 1.87	323.91 ± 47.55	50.35 ± 16.91	280.95 ± 49.13	78.20 ± 30.84
Jeonbuknamwon-1994-3237	185576	MB64	67.33 ± 0.47	Large	0.40 ± 0.05	0.51 ± 0.06	5.52 ± 0.43	327.77 ± 23.85	37.60 ± 7.26	268.46 ± 41.47	48.54 ± 11.84
94WL-12-1	188436	MB65	25.67 ± 0.47	Small	0.62 ± 0.06	0.86 ± 0.10	15.14 ± 0.58	408.68 ± 16.38	110.21 ± 10.64	446.38 ± 16.99	182.14 ± 25.19
VO1538A-GM	189492	MB66	30.67 ± 0.94	Small	0.47 ± 0.01	0.58 ± 0.02	3.67 ± 0.10	251.86 ± 34.17	28.10 ± 6.92	211.60 ± 40.15	39.77 ± 12.06
VO1955B-G	189518	MB67	32.67 ± 0.47	Small	0.26 ± 0.00	0.32 ± 0.00	5.94 ± 1.97	307.37 ± 57.49	42.13 ± 15.03	254.22 ± 68.98	60.69 ± 22.94
VO2018B-BR	189519	MB68	46.33 ± 3.68	Small	0.52 ± 0.03	0.72 ± 0.06	5.86 ± 0.41	311.03 ± 40.64	42.18 ± 3.84	239.42 ± 27.37	58.98 ± 6.31
VO3146B-G	189549	MB69	37.00 ± 0.82	Small	0.34 ± 0.02	0.41 ± 0.02	6.22 ± 1.46	285.62 ± 21.53	29.91 ± 6.30	211.96 ± 25.14	41.82 ± 10.96
VO5040A-G	189563	MB70	28.67 ± 0.47	Small	0.65 ± 0.07	0.85 ± 0.10	7.17 ± 1.99	277.90 ± 52.28	40.75 ± 9.10	257.00 ± 41.91	56.38 ± 16.71
VO5040B-G	189564	MB71	28.33 ± 0.47	Small	0.41 ± 0.02	0.52 ± 0.03	11.38 ± 0.80	340.95 ± 13.13	47.77 ± 0.55	307.77 ± 2.82	70.32 ± 2.25
Gulkeunnokdu	208926	MB72	61.67 ± 1.70	Large	0.30 ± 0.04	0.39 ± 0.05	8.20 ± 1.27	299.47 ± 14.96	34.37 ± 5.00	250.00 ± 29.50	44.97 ± 8.20
Jeonnamwando-2000-112	212099	MB73	47.33 ± 0.47	Small	1.18 ± 0.19	1.58 ± 0.25	8.14 ± 1.26	291.75 ± 36.61	32.15 ± 7.31	227.78 ± 39.55	38.83 ± 12.14
Habcheondokkog-5	219655	MB74	45.33 ± 0.47	Small	0.68 ± 0.11	0.96 ± 0.15	10.11 ± 0.71	335.70 ± 10.61	46.18 ± 1.80	290.49 ± 19.97	65.56 ± 3.76
Jeonnamwando-2008-3	221987	MB75	41.33 ± 0.94	Small	0.34 ± 0.02	0.41 ± 0.03	6.13 ± 0.63	266.89 ± 13.28	24.53 ± 2.44	177.46 ± 7.66	32.30 ± 4.07
PI 362296	225363	MB76	65.00 ± 2.45	Large	0.82 ± 0.10	1.07 ± 0.12	7.47 ± 2.12	285.94 ± 48.53	37.87 ± 11.85	238.50 ± 54.22	54.11 ± 19.98
PI 362319	225377	MB77	37.67 ± 0.47	Small	0.37 ± 0.05	0.46 ± 0.06	8.44 ± 1.24	306.14 ± 32.79	38.86 ± 9.10	247.83 ± 45.84	56.87 ± 16.99
PI 425545	225386	MB78	45.33 ± 0.47	Small	0.37 ± 0.04	0.48 ± 0.06	14.50 ± 1.53	353.51 ± 13.47	47.05 ± 5.61	317.51 ± 28.07	67.45 ± 8.67
PI 425558	225396	MB79	41.00 ± 1.41	Small	0.96 ± 0.11	1.34 ± 0.14	15.45 ± 1.96	340.58 ± 14.43	48.83 ± 1.89	311.18 ± 19.59	67.11 ± 3.30
PI 425563	225401	MB80	39.00 ± 0.00	Small	0.58 ± 0.09	0.73 ± 0.11	9.11 ± 0.97	313.81 ± 37.32	37.11 ± 7.09	253.89 ± 41.36	46.36 ± 13.22
WIR6543	240109	MB81	42.33 ± 1.25	Small	0.34 ± 0.03	0.44 ± 0.04	6.80 ± 0.63	368.27 ± 28.15	60.78 ± 4.80	307.78 ± 8.72	101.98 ± 10.22
Dankukdaehakkyeo-2008-24	242325	MB82	71.33 ± 1.25	Large	0.83 ± 0.01	1.03 ± 0.02	8.27 ± 1.52	315.64 ± 21.68	39.89 ± 5.71	276.59 ± 47.97	51.39 ± 9.10
Anhui hefei 1	242414	MB83	70.00 ± 0.82	Large	0.30 ± 0.04	0.39 ± 0.06	6.53 ± 1.75	254.27 ± 6.74	23.77 ± 2.47	199.65 ± 11.69	27.51 ± 2.37
Anhui hefei 3	242415	MB84	75.33 ± 3.86	Large	0.36 ± 0.01	0.45 ± 0.02	4.81 ± 0.85	215.10 ± 11.42	18.07 ± 1.24	153.49 ± 9.51	20.54 ± 2.61
Anhui hefei 2	250302	MB85	82.33 ± 0.47	Large	0.42 ± 0.05	0.52 ± 0.06	5.57 ± 1.04	226.72 ± 26.48	22.77 ± 4.41	184.66 ± 14.67	24.22 ± 6.57
PI 362313	250355	MB86	43.33 ± 0.47	Small	0.89 ± 0.11	1.26 ± 0.14	9.93 ± 4.25	290.00 ± 57.96	43.32 ± 17.31	252.36 ± 55.68	58.02 ± 22.23

UzRIPI 653	261750	MB87	43.67 ± 0.47	Small	0.54 ± 0.06	0.68 ± 0.08	6.89 ± 2.41	297.61 ± 54.47	46.99 ± 15.01	267.04 ± 59.05	54.05 ± 20.78
UzRIPI 207	261771	MB88	45.33 ± 0.47	Small	0.91 ± 0.14	1.23 ± 0.18	7.38 ± 1.08	265.94 ± 10.96	37.08 ± 3.60	236.53 ± 29.65	36.14 ± 4.48
UzRIPI 185	261773	MB89	36.67 ± 0.47	Small	1.14 ± 0.17	1.49 ± 0.21	7.61 ± 1.40	241.98 ± 31.55	33.09 ± 5.48	223.68 ± 19.27	34.47 ± 7.18
Vo1132	278235	MB90	62.33 ± 0.47	Large	0.33 ± 0.01	0.44 ± 0.02	5.41 ± 0.96	220.07 ± 5.87	19.52 ± 0.51	174.24 ± 19.85	21.11 ± 1.91
JP 31238	278437	MB91	43.67 ± 0.47	Small	0.45 ± 0.05	0.58 ± 0.06	9.83 ± 1.79	265.85 ± 17.65	31.38 ± 7.04	228.43 ± 20.40	37.35 ± 7.19
JP 73333	278438	MB92	56.67 ± 0.47	Medium	0.95 ± 0.09	1.21 ± 0.12	9.51 ± 1.40	296.79 ± 28.24	44.42 ± 11.97	276.72 ± 42.10	58.25 ± 17.41
JP 74746	278440	MB93	74.67 ± 0.47	Large	1.01 ± 0.11	1.22 ± 0.14	4.78 ± 1.62	229.88 ± 11.82	20.99 ± 2.66	163.71 ± 14.77	25.66 ± 3.99
YV 548	278627	MB94	55.00 ± 0.82	Medium	0.24 ± 0.03	0.30 ± 0.03	5.91 ± 0.87	261.02 ± 21.40	36.20 ± 11.39	197.89 ± 20.36	40.63 ± 6.81
CHN-buknongyeon-2011-11	278652	MB95	53.00 ± 1.41	Small	0.30 ± 0.02	0.37 ± 0.02	8.83 ± 0.20	216.39 ± 7.77	27.49 ± 2.01	191.66 ± 4.72	26.93 ± 1.10
Bl07-89	278807	MB96	73.33 ± 0.47	Large	0.33 ± 0.03	0.41 ± 0.04	7.08 ± 0.24	238.54 ± 13.44	26.06 ± 2.54	188.50 ± 12.54	25.20 ± 3.80
Bl89501	278819	MB97	67.67 ± 1.25	Large	0.33 ± 0.05	0.43 ± 0.07	6.77 ± 2.28	264.60 ± 43.07	35.10 ± 10.22	211.82 ± 46.14	38.73 ± 19.84
Bl08-68	278820	MB98	46.00 ± 0.00	Small	0.37 ± 0.03	0.45 ± 0.04	6.33 ± 0.90	258.58 ± 51.50	38.05 ± 15.97	216.34 ± 59.14	44.09 ± 25.08
Bl07-71	278829	MB99	51.33 ± 1.25	Small	0.46 ± 0.05	0.57 ± 0.07	8.43 ± 1.76	276.32 ± 33.21	29.65 ± 9.05	222.80 ± 31.23	34.99 ± 10.25
Bl07-82	278831	MB100	74.67 ± 1.70	Large	0.25 ± 0.01	0.32 ± 0.02	9.26 ± 0.50	254.89 ± 23.22	27.55 ± 4.02	210.70 ± 14.55	31.54 ± 5.84
Bl07-119	278834	MB101	44.33 ± 0.47	Small	0.94 ± 0.07	1.18 ± 0.10	11.44 ± 1.51	327.21 ± 24.02	42.11 ± 6.60	247.60 ± 38.50	47.77 ± 9.71
KPADI 2-213	280275	MB102	42.67 ± 0.47	Small	0.74 ± 0.07	0.95 ± 0.08	9.13 ± 0.63	226.44 ± 4.23	29.55 ± 1.33	211.70 ± 3.44	34.42 ± 2.52
KPADI 2-219	280281	MB103	52.00 ± 0.00	Small	0.33 ± 0.05	0.40 ± 0.06	8.82 ± 0.99	246.00 ± 5.49	32.33 ± 2.19	205.73 ± 6.61	37.35 ± 1.13
KPADI 2-220	280282	MB104	55.33 ± 0.47	Medium	0.44 ± 0.06	0.54 ± 0.08	9.17 ± 0.47	233.51 ± 8.02	26.25 ± 1.90	197.40 ± 7.84	29.64 ± 1.86
CHN-GHC-2003-22	283407	MB105	44.67 ± 0.47	Small	0.70 ± 0.09	0.92 ± 0.12	3.94 ± 0.30	212.32 ± 2.85	28.78 ± 4.72	158.80 ± 1.59	24.71 ± 0.65
DM 005004	286524	MB106	50.00 ± 0.82	Small	0.54 ± 0.01	0.67 ± 0.01	6.82 ± 1.10	240.30 ± 4.99	28.38 ± 1.63	192.43 ± 6.82	23.76 ± 2.73
DM 005019	286529	MB107	55.00 ± 0.00	Medium	0.53 ± 0.02	0.68 ± 0.03	2.73 ± 0.61	218.40 ± 12.27	26.43 ± 0.83	196.12 ± 7.60	25.24 ± 1.65
DM 005024	286531	MB108	53.00 ± 0.82	Small	0.34 ± 0.02	0.43 ± 0.03	7.28 ± 0.97	280.63 ± 29.60	32.84 ± 8.24	241.62 ± 16.79	31.48 ± 6.68
DM 005055	286549	MB109	51.67 ± 0.94	Small	1.13 ± 0.07	1.42 ± 0.09	8.29 ± 2.26	314.55 ± 43.69	52.28 ± 17.51	293.00 ± 59.51	49.16 ± 17.30
VC-2778A	289145	MB110	64.33 ± 0.47	Large	1.33 ± 0.03	1.67 ± 0.03	6.73 ± 0.44	301.22 ± 19.22	52.63 ± 10.63	296.02 ± 29.60	51.12 ± 10.09
DM 005023	290139	MB111	46.67 ± 0.47	Small	0.56 ± 0.08	0.70 ± 0.10	5.56 ± 1.09	256.07 ± 37.83	36.32 ± 15.61	231.77 ± 39.56	33.28 ± 15.24
CHN-2010-20	293904	MB112	55.00 ± 2.83	Medium	0.32 ± 0.01	1.50 ± 0.18	4.69 ± 0.45	254.15 ± 3.75	28.37 ± 0.90	196.88 ± 3.47	24.18 ± 0.67
DM 005015	296750	MB113	39.00 ± 0.00	Small	0.40 ± 0.06	0.40 ± 0.02	3.81 ± 0.64	241.61 ± 3.72	28.37 ± 0.25	205.63 ± 12.66	23.65 ± 1.17
KPADI 2-230	296848	MB114	67.00 ± 0.82	Large	0.22 ± 0.01	0.28 ± 0.02	5.11 ± 0.37	267.11 ± 15.32	31.05 ± 4.69	229.73 ± 13.43	27.89 ± 5.38
KPADI 2-236	296852	MB115	47.00 ± 0.00	Small	0.24 ± 0.03	0.30 ± 0.04	4.46 ± 0.60	250.87 ± 5.58	31.48 ± 2.59	203.05 ± 3.89	27.52 ± 2.66
KPADI 2-254	296863	MB116	62.67 ± 0.47	Large	0.93 ± 0.11	1.20 ± 0.14	5.94 ± 0.58	261.49 ± 15.42	35.09 ± 5.88	234.84 ± 23.55	28.41 ± 4.83

KPADI 2-277	296884	MB117	77.00 ± 5.72	Large	0.68 ± 0.06	0.90 ± 0.08	4.80 ± 1.44	284.51 ± 20.30	42.33 ± 10.06	257.93 ± 28.80	36.09 ± 9.00
KPADI 2-288	296895	MB118	63.33 ± 0.47	Large	0.37 ± 0.05	0.47 ± 0.07	4.35 ± 1.73	252.79 ± 18.10	32.22 ± 5.99	226.40 ± 17.49	27.46 ± 5.13
KPADI 2-299	296905	MB119	56.33 ± 0.94	Medium	0.26 ± 0.03	0.32 ± 0.03	4.07 ± 1.15	256.72 ± 22.88	38.31 ± 7.79	232.62 ± 26.17	34.92 ± 9.06
KPADI 2-300	296906	MB120	56.33 ± 0.47	Medium	0.71 ± 0.04	0.95 ± 0.05	4.32 ± 0.83	263.69 ± 9.01	35.13 ± 2.95	245.75 ± 8.01	30.48 ± 2.41
KPADI 2-306	296912	MB121	67.33 ± 0.47	Large	0.57 ± 0.07	0.73 ± 0.10	3.95 ± 0.57	278.53 ± 14.89	40.07 ± 4.61	246.62 ± 16.79	33.18 ± 5.27
KPADI 2-315	296921	MB122	63.33 ± 2.05	Large	0.34 ± 0.05	0.43 ± 0.06	4.46 ± 0.69	267.62 ± 22.53	37.15 ± 6.45	220.25 ± 28.45	28.20 ± 6.65
KPADI 2-322	296928	MB123	66.33 ± 0.47	Large	0.85 ± 0.12	1.08 ± 0.16	2.81 ± 0.68	227.80 ± 5.04	26.44 ± 2.53	213.08 ± 45.35	20.90 ± 1.69
KPADI 2-328	296934	MB124	51.33 ± 0.47	Small	0.30 ± 0.00	0.39 ± 0.01	5.17 ± 0.60	266.27 ± 4.83	29.42 ± 0.86	209.73 ± 4.77	25.24 ± 1.18
Gyeonggiyeoju-2013-63	297537	MB125	55.33 ± 0.47	Medium	0.41 ± 0.05	0.50 ± 0.06	4.38 ± 2.05	265.47 ± 42.89	36.42 ± 13.29	206.38 ± 40.42	30.69 ± 11.52
KPADI 2-346	310362	MB126	69.00 ± 0.82	Large	0.23 ± 0.01	0.30 ± 0.01	6.31 ± 0.21	243.06 ± 5.58	25.04 ± 1.38	186.87 ± 4.83	20.16 ± 1.84
KPADI 2-377	310393	MB127	68.67 ± 0.47	Large	0.72 ± 0.09	0.96 ± 0.13	7.19 ± 0.75	240.91 ± 12.94	30.04 ± 4.99	211.36 ± 15.30	23.44 ± 3.72
KPADI 2-393	310409	MB128	71.33 ± 0.47	Large	0.25 ± 0.03	0.32 ± 0.04	6.93 ± 0.20	245.49 ± 8.93	34.48 ± 2.07	188.17 ± 4.55	27.67 ± 1.06
KPADI 2-403	310419	MB129	68.33 ± 0.47	Large	0.66 ± 0.08	0.91 ± 0.12	9.47 ± 1.16	310.30 ± 40.24	44.64 ± 14.13	222.81 ± 42.36	35.03 ± 13.45
KPADI 2-409	310425	MB130	70.00 ± 0.82	Large	0.21 ± 0.00	0.28 ± 0.00	8.66 ± 2.24	275.58 ± 40.88	46.28 ± 17.84	241.32 ± 52.24	38.52 ± 16.86
KPADI 2-426	310441	MB131	82.33 ± 3.30	Large	0.36 ± 0.00	0.45 ± 0.00	6.93 ± 1.81	257.10 ± 43.80	38.21 ± 15.88	229.50 ± 86.41	32.07 ± 13.74
KPADI 2-432	310445	MB132	56.33 ± 2.36	Medium	0.66 ± 0.06	0.88 ± 0.09	9.39 ± 1.46	268.23 ± 25.15	44.11 ± 8.54	242.84 ± 27.82	36.67 ± 8.29
KPADI 2-434	310447	MB133	45.67 ± 0.47	Small	0.44 ± 0.01	0.56 ± 0.00	8.48 ± 0.35	254.19 ± 12.55	39.38 ± 2.53	228.17 ± 7.41	33.97 ± 3.06
KPADI 2-436	310449	MB134	70.33 ± 1.70	Large	0.25 ± 0.02	0.31 ± 0.02	6.97 ± 0.74	278.01 ± 14.40	42.75 ± 4.01	229.85 ± 11.44	32.91 ± 3.26
KPADI 2-450	310463	MB135	55.00 ± 0.82	Medium	0.22 ± 0.03	0.29 ± 0.04	9.15 ± 1.46	296.91 ± 23.92	49.49 ± 15.33	242.56 ± 39.30	40.16 ± 13.49
KPADI 2-453	310466	MB136	40.33 ± 0.47	Small	0.26 ± 0.02	0.32 ± 0.03	8.28 ± 0.79	282.88 ± 15.47	45.45 ± 7.04	257.84 ± 21.42	34.45 ± 6.30
Range			23.67-82.33		0.21-1.33	0.26-1.67	1.35-34.56	1.66-4.09	12.57-110.21	81.96-446.38	18.45-182.14
Total mean			49.73		0.50	0.66	7.37	2.74	36.66	227.93	42.92
Total SD			13.38		0.21	0.34	4.25	0.41	12.52	51.23	19.98
Total CV			26.90		48.41	51.59	57.64	15.12	34.15	22.47	46.55
p-value			<0.0001		<0.0001	<0.0001	<0.0001	<0.0001	<0.0001	<0.0001	<0.0001

Table S2. Nutritional and fatty acid contents of 136 mung bean accessions.

Code	Total nutritional contents (g/100g)		Fatty acid contents (g/100g)							ω-6:ω-3 ratio	DBI
	**TP**	**TS**	PA	SA	OA	LA	LLA	TSFA	TUFA		
**MB1**	25.26 ± 0.05	47.90 ± 0.44	29.95 ± 0.05	5.19 ± 0.02	2.90 ± 0.05	45.46 ± 0.05	16.51 ± 0.07	35.14 ± 0.04	64.46 ± 0.04	2.75	143.33
**MB2**	24.87 ± 0.12	41.79 ± 0.48	30.31 ± 0.03	4.99 ± 0.01	3.00 ± 0.08	44.84 ± 0.05	16.86 ± 0.04	35.31 ± 0.04	64.46 ± 0.04	2.66	143.25
**MB3**	25.28 ± 0.12	48.25 ± 0.36	29.25 ± 0.03	5.91 ± 0.05	2.86 ± 0.04	45.04 ± 0.04	16.95 ± 0.04	35.16 ± 0.07	64.46 ± 0.07	2.66	143.77
**MB4**	26.72 ± 0.24	43.36 ± 0.11	30.18 ± 0.04	4.72 ± 0.01	2.21 ± 0.06	46.61 ± 0.03	16.27 ± 0.02	34.91 ± 0.04	65.46 ± 0.04	2.86	144.25
**MB5**	25.67 ± 0.06	45.05 ± 0.46	29.81 ± 0.03	4.52 ± 0.02	2.92 ± 0.07	45.30 ± 0.09	17.46 ± 0.04	34.32 ± 0.04	65.46 ± 0.04	2.59	145.9
**MB6**	27.21 ± 0.13	40.73 ± 0.12	28.16 ± 0.01	5.15 ± 0.08	2.10 ± 0.02	44.76 ± 0.06	19.83 ± 0.03	33.31 ± 0.08	66.46 ± 0.08	2.26	151.11
**MB7**	25.29 ± 0.14	49.03 ± 0.31	29.80 ± 0.05	4.18 ± 0.01	2.00 ± 0.01	46.38 ± 0.00	17.64 ± 0.03	33.98 ± 0.04	66.46 ± 0.04	2.63	147.69
**MB8**	25.12 ± 0.01	42.49 ± 0.10	29.26 ± 0.02	5.15 ± 0.10	2.77 ± 0.02	46.78 ± 0.08	16.05 ± 0.02	34.41 ± 0.08	65.46 ± 0.08	2.91	144.47
**MB9**	23.90 ± 0.09	43.64 ± 0.40	29.53 ± 0.03	5.01 ± 0.08	3.04 ± 0.05	45.19 ± 0.07	17.24 ± 0.03	34.54 ± 0.07	65.46 ± 0.06	2.62	145.12
**MB10**	25.65 ± 0.12	42.91 ± 0.50	30.28 ± 0.02	4.14 ± 0.01	2.45 ± 0.03	44.00 ± 0.01	19.13 ± 0.03	34.42 ± 0.01	65.46 ± 0.01	2.30	147.85
**MB11**	26.12 ± 0.10	40.96 ± 0.26	30.59 ± 0.04	4.86 ± 0.08	2.50 ± 0.03	46.75 ± 0.05	15.30 ± 0.01	35.45 ± 0.04	64.46 ± 0.04	3.06	141.9
**MB12**	27.10 ± 0.02	43.19 ± 0.44	29.77 ± 0.03	4.43 ± 0.12	2.44 ± 0.03	46.71 ± 0.07	16.66 ± 0.04	34.20 ± 0.09	65.46 ± 0.09	2.80	145.82
**MB13**	26.54 ± 0.10	44.67 ± 0.42	29.97 ± 0.00	4.38 ± 0.07	2.48 ± 0.02	45.75 ± 0.08	17.42 ± 0.04	34.35 ± 0.07	65.46 ± 0.07	2.63	146.25
**MB14**	26.27 ± 0.20	38.45 ± 0.18	30.62 ± 0.08	4.06 ± 0.05	2.23 ± 0.08	47.53 ± 0.04	15.56 ± 0.03	34.68 ± 0.07	65.46 ± 0.07	3.05	143.97
**MB15**	27.17 ± 0.10	45.79 ± 0.10	30.29 ± 0.00	4.89 ± 0.06	2.36 ± 0.03	47.49 ± 0.08	14.97 ± 0.04	35.18 ± 0.06	64.46 ± 0.06	3.17	142.25
**MB16**	23.29 ± 0.17	43.23 ± 0.38	29.65 ± 0.09	4.38 ± 0.01	2.30 ± 0.00	44.72 ± 0.06	18.95 ± 0.02	34.03 ± 0.09	65.46 ± 0.09	2.36	148.6
**MB17**	26.19 ± 0.15	46.55 ± 0.39	30.96 ± 0.03	5.24 ± 0.03	2.81 ± 0.01	38.30 ± 0.07	22.69 ± 0.06	36.20 ± 0.02	63.46 ± 0.02	1.69	147.48
**MB18**	27.45 ± 0.22	39.45 ± 0.49	30.80 ± 0.02	4.67 ± 0.01	2.46 ± 0.03	39.52 ± 0.03	22.55 ± 0.03	35.47 ± 0.03	64.46 ± 0.03	1.75	149.15
**MB19**	26.37 ± 0.19	39.12 ± 0.16	29.98 ± 0.11	4.61 ± 0.20	1.94 ± 0.03	46.58 ± 0.06	16.90 ± 0.06	34.59 ± 0.09	65.46 ± 0.09	2.76	145.79
**MB20**	25.40 ± 0.11	38.81 ± 0.47	30.12 ± 0.03	4.61 ± 0.03	1.93 ± 0.02	46.00 ± 0.07	17.34 ± 0.04	34.73 ± 0.03	65.46 ± 0.03	2.65	145.94
**MB21**	26.88 ± 0.06	42.54 ± 0.40	29.53 ± 0.01	4.36 ± 0.01	2.01 ± 0.09	47.85 ± 0.06	16.24 ± 0.04	33.89 ± 0.00	66.46 ± 0.00	2.95	146.44
**MB22**	28.55 ± 0.10	40.22 ± 0.49	29.08 ± 0.07	4.56 ± 0.12	2.11 ± 0.10	48.14 ± 0.31	16.10 ± 0.03	33.64 ± 0.19	66.46 ± 0.19	2.99	146.71
**MB23**	24.94 ± 0.10	41.41 ± 0.22	30.39 ± 0.07	4.53 ± 0.01	1.96 ± 0.08	48.88 ± 0.04	14.25 ± 0.03	34.91 ± 0.07	65.46 ± 0.07	3.43	142.46
**MB24**	25.25 ± 0.02	45.13 ± 0.45	29.03 ± 0.05	4.23 ± 0.06	1.65 ± 0.03	46.71 ± 0.10	18.38 ± 0.05	33.26 ± 0.11	66.46 ± 0.11	2.54	150.21
**MB25**	24.30 ± 0.07	42.30 ± 0.17	29.34 ± 0.03	5.25 ± 0.00	2.15 ± 0.05	49.09 ± 0.03	14.18 ± 0.02	34.58 ± 0.02	65.46 ± 0.02	3.46	142.86
**MB26**	25.18 ± 0.08	47.40 ± 0.22	29.26 ± 0.04	4.24 ± 0.03	2.37 ± 0.01	44.89 ± 0.10	19.23 ± 0.04	33.51 ± 0.07	66.46 ± 0.07	2.33	149.84

**MB27**	25.61 ± 0.12	40.48 ± 0.36	29.59 ± 0.04	4.42 ± 0.02	2.49 ± 0.04	46.79 ± 0.02	16.71 ± 0.01	34.01 ± 0.02	65.46 ± 0.02	2.80	146.21
**MB28**	25.79 ± 0.03	45.13 ± 0.32	29.45 ± 0.20	5.01 ± 0.02	2.51 ± 0.02	46.18 ± 0.13	16.85 ± 0.04	34.47 ± 0.19	65.46 ± 0.19	2.74	145.41
**MB29**	27.06 ± 0.12	42.27 ± 0.39	30.17 ± 0.12	4.64 ± 0.01	2.35 ± 0.08	41.31 ± 0.09	21.53 ± 0.04	34.81 ± 0.12	65.46 ± 0.12	1.92	149.57
**MB30**	26.30 ± 0.04	41.29 ± 0.17	30.32 ± 0.03	4.21 ± 0.01	2.34 ± 0.04	46.02 ± 0.03	17.10 ± 0.01	34.53 ± 0.03	65.46 ± 0.03	2.69	145.7
**MB31**	24.15 ± 0.16	42.58 ± 0.20	31.32 ± 0.05	5.08 ± 0.04	2.32 ± 0.05	43.72 ± 0.04	17.55 ± 0.02	36.40 ± 0.02	63.46 ± 0.02	2.49	142.43
**MB32**	25.23 ± 0.22	38.60 ± 0.27	30.62 ± 0.03	4.67 ± 0.01	2.28 ± 0.01	44.82 ± 0.03	17.62 ± 0.03	35.29 ± 0.02	64.46 ± 0.02	2.54	144.76
**MB33**	25.46 ± 0.05	36.82 ± 0.25	29.74 ± 0.04	4.99 ± 0.02	2.34 ± 0.03	44.64 ± 0.01	18.29 ± 0.02	34.73 ± 0.04	65.46 ± 0.03	2.44	146.49
**MB34**	28.15 ± 0.08	35.09 ± 0.35	30.72 ± 0.02	4.36 ± 0.03	2.48 ± 0.02	39.19 ± 0.06	23.26 ± 0.03	35.08 ± 0.04	64.46 ± 0.04	1.68	150.61
**MB35**	23.59 ± 0.06	38.65 ± 0.23	31.83 ± 0.07	3.65 ± 0.00	2.20 ± 0.02	46.12 ± 0.06	16.20 ± 0.01	35.49 ± 0.07	64.46 ± 0.07	2.85	143.03
**MB36**	25.62 ± 0.13	40.11 ± 0.49	30.32 ± 0.04	4.93 ± 0.01	2.93 ± 0.04	46.78 ± 0.01	15.03 ± 0.03	35.26 ± 0.04	64.46 ± 0.04	3.11	141.59
**MB37**	26.59 ± 0.05	37.88 ± 0.25	30.89 ± 0.07	4.72 ± 0.06	2.09 ± 0.19	44.37 ± 0.12	17.94 ± 0.06	35.60 ± 0.03	64.46 ± 0.03	2.47	144.65
**MB38**	23.76 ± 0.03	38.52 ± 0.08	29.68 ± 0.04	4.56 ± 0.03	2.75 ± 0.03	44.35 ± 0.06	18.67 ± 0.02	34.24 ± 0.05	65.46 ± 0.05	2.38	147.44
**MB39**	27.18 ± 0.10	38.83 ± 0.45	30.94 ± 0.06	5.62 ± 0.02	2.64 ± 0.01	44.83 ± 0.05	15.97 ± 0.02	36.56 ± 0.04	63.46 ± 0.04	2.81	140.21
**MB40**	26.36 ± 0.08	39.13 ± 0.50	30.17 ± 0.02	5.10 ± 0.02	2.35 ± 0.01	45.23 ± 0.02	17.15 ± 0.04	35.27 ± 0.04	64.46 ± 0.04	2.64	144.25
**MB41**	26.67 ± 0.10	40.06 ± 0.40	29.83 ± 0.08	4.82 ± 0.08	2.82 ± 0.08	42.63 ± 0.12	19.91 ± 0.04	34.64 ± 0.08	65.46 ± 0.08	2.14	147.8
**MB42**	26.99 ± 0.07	38.02 ± 0.26	29.34 ± 0.03	4.93 ± 0.02	2.72 ± 0.01	45.01 ± 0.05	18.00 ± 0.02	34.27 ± 0.06	65.46 ± 0.06	2.50	146.73
**MB43**	25.48 ± 0.25	38.39 ± 0.22	30.74 ± 0.04	4.74 ± 0.03	1.80 ± 0.02	42.21 ± 0.06	20.52 ± 0.03	35.48 ± 0.06	64.46 ± 0.06	2.06	147.77
**MB44**	25.91 ± 0.06	38.71 ± 0.41	30.58 ± 0.03	4.88 ± 0.05	2.57 ± 0.01	45.53 ± 0.03	16.45 ± 0.02	35.46 ± 0.03	64.46 ± 0.03	2.77	142.96
**MB45**	25.54 ± 0.07	41.79 ± 0.06	30.18 ± 0.03	4.71 ± 0.07	2.48 ± 0.00	45.41 ± 0.08	17.23 ± 0.02	34.88 ± 0.06	65.46 ± 0.06	2.64	144.98
**MB46**	24.80 ± 0.10	42.95 ± 0.49	30.09 ± 0.07	4.55 ± 0.05	2.25 ± 0.17	45.54 ± 0.11	17.57 ± 0.03	34.64 ± 0.05	65.46 ± 0.05	2.59	146.04
**MB47**	23.81 ± 0.05	37.43 ± 0.26	29.95 ± 0.02	3.82 ± 0.03	2.73 ± 0.01	44.92 ± 0.01	18.57 ± 0.02	33.77 ± 0.03	66.46 ± 0.02	2.42	148.29
**MB48**	22.71 ± 0.04	46.08 ± 0.24	29.05 ± 0.05	5.18 ± 0.02	2.67 ± 0.02	45.06 ± 0.05	18.05 ± 0.06	34.23 ± 0.03	65.46 ± 0.03	2.50	146.93
**MB49**	25.62 ± 0.14	40.17 ± 0.37	29.72 ± 0.04	5.00 ± 0.02	2.80 ± 0.02	46.08 ± 0.06	16.40 ± 0.03	34.71 ± 0.02	65.46 ± 0.02	2.81	144.18
**MB50**	23.25 ± 0.04	43.28 ± 0.49	29.91 ± 0.03	4.74 ± 0.10	2.51 ± 0.04	46.49 ± 0.27	16.35 ± 0.12	34.65 ± 0.13	65.46 ± 0.13	2.84	144.55
**MB51**	23.29 ± 0.10	43.31 ± 0.24	30.72 ± 0.03	4.44 ± 0.02	2.83 ± 0.00	45.07 ± 0.04	16.94 ± 0.07	35.16 ± 0.04	64.46 ± 0.04	2.66	143.8
**MB52**	25.56 ± 0.07	41.46 ± 0.45	29.57 ± 0.07	5.65 ± 0.02	3.15 ± 0.04	45.45 ± 0.01	16.18 ± 0.05	35.22 ± 0.06	64.46 ± 0.06	2.81	142.59
**MB53**	25.80 ± 0.04	42.08 ± 0.48	28.86 ± 0.03	4.20 ± 0.01	2.91 ± 0.02	46.34 ± 0.02	17.69 ± 0.03	33.06 ± 0.03	66.46 ± 0.04	2.62	148.66
**MB54**	25.43 ± 0.15	43.32 ± 0.30	29.82 ± 0.11	5.77 ± 0.03	2.42 ± 0.06	47.09 ± 0.02	14.90 ± 0.05	35.59 ± 0.07	64.46 ± 0.07	3.16	141.3
**MB55**	24.49 ± 0.08	38.63 ± 0.27	29.95 ± 0.05	5.20 ± 0.01	2.38 ± 0.03	47.40 ± 0.05	15.06 ± 0.01	35.16 ± 0.06	64.46 ± 0.06	3.15	142.37
**MB56**	28.96 ± 0.05	36.29 ± 0.43	30.38 ± 0.05	4.37 ± 0.08	2.46 ± 0.01	39.13 ± 0.05	23.66 ± 0.04	34.75 ± 0.03	65.46 ± 0.03	1.65	151.71

**MB57**	24.72 ± 0.18	39.25 ± 0.41	29.39 ± 0.03	2.95 ± 0.12	1.75 ± 0.02	44.51 ± 0.06	21.40 ± 0.05	32.34 ± 0.10	67.46 ± 0.10	2.08	154.98
**MB58**	26.89 ± 0.09	39.14 ± 0.39	30.35 ± 0.05	5.06 ± 0.13	2.34 ± 0.00	46.29 ± 0.02	15.97 ± 0.08	35.41 ± 0.09	64.46 ± 0.09	2.90	142.81
**MB59**	26.10 ± 0.15	37.22 ± 0.10	30.40 ± 0.03	4.71 ± 0.02	2.27 ± 0.01	46.06 ± 0.02	16.56 ± 0.03	35.11 ± 0.02	64.46 ± 0.02	2.78	144.07
**MB60**	25.99 ± 0.05	37.70 ± 0.14	30.49 ± 0.00	4.77 ± 0.03	2.69 ± 0.05	46.27 ± 0.05	15.78 ± 0.02	35.26 ± 0.03	64.46 ± 0.03	2.93	142.57
**MB61**	24.25 ± 0.07	37.88 ± 0.42	30.67 ± 0.03	4.23 ± 0.06	2.25 ± 0.02	37.96 ± 0.02	24.89 ± 0.03	34.90 ± 0.03	65.46 ± 0.03	1.53	152.83
**MB62**	25.45 ± 0.03	40.83 ± 0.44	30.57 ± 0.03	3.65 ± 0.05	1.95 ± 0.03	46.79 ± 0.04	17.04 ± 0.02	34.22 ± 0.08	65.46 ± 0.08	2.74	146.65
**MB63**	27.99 ± 0.06	36.94 ± 0.07	30.55 ± 0.07	4.07 ± 0.09	2.20 ± 0.01	45.62 ± 0.05	17.55 ± 0.06	34.62 ± 0.08	65.46 ± 0.08	2.60	146.1
**MB64**	26.45 ± 0.09	41.79 ± 0.48	29.67 ± 0.02	2.67 ± 0.03	2.06 ± 0.06	46.57 ± 0.12	19.03 ± 0.09	32.34 ± 0.02	67.46 ± 0.02	2.45	152.29
**MB65**	26.17 ± 0.10	33.01 ± 0.49	31.13 ± 0.01	3.62 ± 0.03	2.05 ± 0.02	46.50 ± 0.02	16.69 ± 0.02	34.75 ± 0.04	65.46 ± 0.04	2.79	145.14
**MB66**	24.43 ± 0.05	40.37 ± 0.25	30.15 ± 0.09	2.49 ± 0.06	2.82 ± 0.00	46.51 ± 0.05	18.03 ± 0.10	32.64 ± 0.14	67.46 ± 0.14	2.58	149.93
**MB67**	26.36 ± 0.22	36.43 ± 0.40	29.45 ± 0.06	2.41 ± 0.15	2.52 ± 0.01	46.85 ± 0.05	18.77 ± 0.04	31.86 ± 0.09	68.46 ± 0.09	2.50	152.53
**MB68**	24.64 ± 0.06	32.62 ± 0.49	29.45 ± 0.02	2.85 ± 0.00	2.37 ± 0.01	44.54 ± 0.02	20.79 ± 0.02	32.30 ± 0.02	67.46 ± 0.02	2.14	153.8
**MB69**	26.80 ± 0.08	42.53 ± 0.39	30.62 ± 0.78	2.44 ± 0.13	2.33 ± 0.10	47.93 ± 0.32	16.68 ± 0.69	33.06 ± 0.90	66.46 ± 0.91	2.87	148.23
**MB70**	27.77 ± 0.06	34.20 ± 0.49	30.55 ± 0.09	2.36 ± 0.20	2.32 ± 0.01	49.40 ± 0.10	15.37 ± 0.02	32.91 ± 0.11	67.46 ± 0.11	3.21	147.24
**MB71**	25.79 ± 0.02	35.69 ± 0.26	27.71 ± 0.52	4.91 ± 1.98	2.17 ± 0.26	47.24 ± 0.89	17.98 ± 0.31	32.62 ± 1.46	67.46 ± 1.46	2.63	150.57
**MB72**	26.90 ± 0.11	42.12 ± 0.43	30.00 ± 0.14	2.51 ± 0.50	2.44 ± 0.01	47.32 ± 0.26	17.73 ± 0.10	32.51 ± 0.36	67.46 ± 0.36	2.67	150.26
**MB73**	26.98 ± 0.18	40.65 ± 0.34	30.56 ± 0.01	2.61 ± 0.03	2.01 ± 0.02	46.20 ± 0.07	18.62 ± 0.04	33.17 ± 0.03	66.46 ± 0.03	2.48	150.27
**MB74**	24.56 ± 0.13	42.89 ± 0.20	31.11 ± 0.03	2.28 ± 0.05	2.06 ± 0.01	46.84 ± 0.02	17.72 ± 0.01	33.38 ± 0.04	66.46 ± 0.04	2.64	148.89
**MB75**	24.53 ± 0.07	42.14 ± 0.19	29.06 ± 0.04	7.01 ± 0.02	1.95 ± 0.02	45.00 ± 0.04	16.99 ± 0.01	36.07 ± 0.05	63.46 ± 0.05	2.65	142.9
**MB76**	28.11 ± 0.11	40.16 ± 0.24	29.48 ± 0.03	1.99 ± 0.03	2.08 ± 0.02	49.66 ± 0.03	16.78 ± 0.02	31.47 ± 0.01	68.46 ± 0.01	2.96	151.76
**MB77**	24.80 ± 0.02	37.09 ± 0.20	30.75 ± 0.05	2.74 ± 0.20	2.31 ± 0.06	46.93 ± 0.16	17.27 ± 0.03	33.49 ± 0.15	66.46 ± 0.14	2.72	147.97
**MB78**	24.23 ± 0.06	44.61 ± 0.47	30.75 ± 0.04	2.33 ± 0.02	2.79 ± 0.03	46.76 ± 0.02	17.37 ± 0.03	33.08 ± 0.06	66.46 ± 0.06	2.69	148.42
**MB79**	26.59 ± 0.12	38.40 ± 0.50	30.44 ± 0.04	2.46 ± 0.05	2.78 ± 0.01	40.93 ± 0.01	23.39 ± 0.02	32.90 ± 0.01	67.46 ± 0.01	1.75	154.8
**MB80**	26.16 ± 0.20	40.62 ± 0.30	29.97 ± 0.01	2.02 ± 0.03	2.59 ± 0.02	48.68 ± 0.04	16.74 ± 0.06	31.99 ± 0.02	68.46 ± 0.02	2.91	150.17
**MB81**	24.74 ± 0.05	38.03 ± 0.02	30.68 ± 0.14	2.12 ± 0.48	2.27 ± 0.02	47.49 ± 0.22	17.43 ± 0.10	32.81 ± 0.34	67.46 ± 0.34	2.72	149.55
**MB82**	24.75 ± 0.10	42.17 ± 0.18	29.27 ± 0.10	2.46 ± 0.35	1.83 ± 0.02	49.10 ± 0.18	17.34 ± 0.08	31.73 ± 0.25	68.46 ± 0.25	2.83	152.04
**MB83**	23.85 ± 0.07	45.64 ± 0.45	30.06 ± 0.06	2.48 ± 0.25	2.11 ± 0.02	49.65 ± 0.10	15.70 ± 0.07	32.54 ± 0.19	67.46 ± 0.19	3.16	148.51
**MB84**	26.24 ± 0.14	40.15 ± 0.25	30.49 ± 0.06	2.04 ± 0.15	2.44 ± 0.03	50.08 ± 0.05	14.95 ± 0.03	32.53 ± 0.10	67.46 ± 0.10	3.35	147.46
**MB85**	25.20 ± 0.02	41.08 ± 0.28	30.29 ± 0.01	1.62 ± 0.09	2.24 ± 0.02	50.71 ± 0.10	15.15 ± 0.06	31.91 ± 0.08	68.46 ± 0.08	3.35	149.1
**MB86**	25.16 ± 0.09	37.85 ± 0.26	30.28 ± 0.04	2.01 ± 0.14	2.09 ± 0.02	49.57 ± 0.07	16.05 ± 0.03	32.29 ± 0.11	67.46 ± 0.11	3.09	149.38

**MB87**	26.91 ± 0.20	39.25 ± 0.17	30.75 ± 0.05	1.84 ± 0.13	2.35 ± 0.00	48.62 ± 0.06	16.44 ± 0.03	32.59 ± 0.08	67.46 ± 0.08	2.96	148.91
**MB88**	24.53 ± 0.08	43.93 ± 0.12	30.50 ± 0.06	1.86 ± 0.15	2.18 ± 0.03	47.54 ± 0.05	17.92 ± 0.03	32.36 ± 0.11	67.46 ± 0.11	2.65	151.02
**MB89**	24.90 ± 0.24	37.07 ± 0.40	29.81 ± 0.05	1.92 ± 0.25	1.62 ± 0.01	48.00 ± 0.19	18.65 ± 0.03	31.73 ± 0.20	68.46 ± 0.20	2.57	153.57
**MB90**	26.02 ± 0.08	40.92 ± 0.33	30.04 ± 0.16	2.00 ± 0.07	3.01 ± 0.43	47.57 ± 0.17	17.38 ± 0.37	32.05 ± 0.10	67.46 ± 0.10	2.74	150.28
**MB91**	26.14 ± 0.07	39.51 ± 0.28	30.64 ± 0.25	2.78 ± 0.29	3.13 ± 0.47	45.38 ± 0.39	18.08 ± 0.11	33.42 ± 0.06	66.46 ± 0.06	2.51	148.12
**MB92**	25.62 ± 0.06	37.40 ± 0.07	30.23 ± 0.07	2.43 ± 0.15	2.08 ± 0.02	48.21 ± 0.08	17.05 ± 0.02	32.66 ± 0.09	67.46 ± 0.09	2.83	149.65
**MB93**	28.37 ± 0.09	40.53 ± 0.43	29.59 ± 0.03	2.42 ± 0.20	2.06 ± 0.02	46.77 ± 0.11	19.17 ± 0.07	32.00 ± 0.18	68.46 ± 0.18	2.44	153.1
**MB94**	25.08 ± 0.03	40.85 ± 0.41	30.38 ± 0.07	2.67 ± 0.29	1.76 ± 0.04	49.07 ± 0.14	16.12 ± 0.04	33.05 ± 0.22	66.46 ± 0.22	3.04	148.27
**MB95**	27.77 ± 0.04	38.60 ± 0.23	29.70 ± 0.09	2.82 ± 0.38	1.63 ± 0.02	47.86 ± 0.25	18.00 ± 0.04	32.52 ± 0.30	67.46 ± 0.30	2.66	151.34
**MB96**	25.05 ± 0.04	39.88 ± 0.31	31.19 ± 0.02	2.55 ± 0.02	1.87 ± 0.02	47.55 ± 0.03	16.84 ± 0.03	33.74 ± 0.03	66.46 ± 0.03	2.82	147.5
**MB97**	25.50 ± 0.27	39.86 ± 0.37	30.41 ± 0.05	2.28 ± 0.15	1.74 ± 0.06	48.07 ± 0.12	17.50 ± 0.03	32.69 ± 0.18	67.46 ± 0.18	2.75	150.4
**MB98**	25.79 ± 0.03	39.40 ± 0.42	30.21 ± 0.04	2.06 ± 0.02	2.22 ± 0.02	50.67 ± 0.04	14.85 ± 0.01	32.27 ± 0.06	67.46 ± 0.06	3.41	148.09
**MB99**	27.59 ± 0.15	42.40 ± 0.20	30.02 ± 0.04	2.52 ± 0.01	2.49 ± 0.03	48.43 ± 0.02	16.53 ± 0.01	32.55 ± 0.05	67.46 ± 0.05	2.93	148.95
**MB100**	24.90 ± 0.10	41.09 ± 0.14	30.58 ± 0.09	2.39 ± 0.30	3.10 ± 0.01	48.22 ± 0.14	15.70 ± 0.06	32.98 ± 0.21	67.46 ± 0.21	3.07	146.66
**MB101**	27.25 ± 0.13	42.32 ± 0.34	29.32 ± 0.60	3.72 ± 2.03	2.21 ± 0.04	47.88 ± 1.04	16.87 ± 0.37	33.04 ± 1.43	66.46 ± 1.43	2.84	148.58
**MB102**	26.25 ± 0.11	37.77 ± 0.49	30.03 ± 0.12	2.41 ± 0.43	2.18 ± 0.03	50.35 ± 0.22	15.03 ± 0.06	32.44 ± 0.31	67.46 ± 0.31	3.35	147.97
**MB103**	27.47 ± 0.04	39.35 ± 0.42	29.86 ± 0.07	2.10 ± 0.21	2.23 ± 0.04	48.40 ± 0.15	17.42 ± 0.02	31.95 ± 0.17	68.46 ± 0.17	2.78	151.3
**MB104**	25.70 ± 0.10	41.22 ± 0.37	29.88 ± 0.10	2.58 ± 0.21	2.20 ± 0.02	48.16 ± 0.08	17.17 ± 0.03	32.46 ± 0.13	67.46 ± 0.13	2.81	150.04
**MB105**	26.41 ± 0.03	40.17 ± 0.15	31.00 ± 0.21	2.71 ± 0.41	2.37 ± 0.02	47.37 ± 0.17	16.56 ± 0.05	33.70 ± 0.20	66.46 ± 0.20	2.86	146.78
**MB106**	26.93 ± 0.09	39.89 ± 0.25	30.66 ± 0.19	2.86 ± 0.42	2.71 ± 0.00	47.81 ± 0.21	15.95 ± 0.07	33.52 ± 0.27	66.46 ± 0.28	3.00	146.2
**MB107**	25.97 ± 0.11	38.05 ± 0.45	29.78 ± 0.08	2.98 ± 0.28	2.19 ± 0.02	50.21 ± 0.13	14.83 ± 0.06	32.76 ± 0.22	67.46 ± 0.22	3.38	147.12
**MB108**	26.64 ± 0.02	37.88 ± 0.38	30.93 ± 0.04	3.53 ± 0.03	1.72 ± 0.03	46.98 ± 0.04	16.85 ± 0.02	34.45 ± 0.06	65.46 ± 0.06	2.79	146.23
**MB109**	27.18 ± 0.08	40.07 ± 0.36	30.58 ± 0.22	2.88 ± 0.58	2.01 ± 0.03	47.83 ± 0.26	16.71 ± 0.09	33.46 ± 0.36	66.46 ± 0.36	2.86	147.79
**MB110**	24.25 ± 0.15	43.89 ± 0.28	31.59 ± 0.12	2.16 ± 0.22	1.62 ± 0.01	47.45 ± 0.11	17.17 ± 0.02	33.76 ± 0.13	66.46 ± 0.13	2.76	148.03
**MB111**	26.74 ± 0.06	39.68 ± 0.17	30.39 ± 0.23	3.21 ± 0.67	2.01 ± 0.04	46.66 ± 0.29	17.73 ± 0.11	33.60 ± 0.44	66.46 ± 0.44	2.63	148.52
**MB112**	25.81 ± 0.08	39.26 ± 0.49	30.10 ± 0.20	3.38 ± 0.63	2.03 ± 0.03	48.04 ± 0.31	16.45 ± 0.10	33.48 ± 0.44	66.46 ± 0.44	2.92	147.46
**MB113**	24.90 ± 0.05	39.00 ± 0.42	31.94 ± 0.15	2.49 ± 0.58	2.13 ± 0.04	46.51 ± 0.30	16.94 ± 0.10	34.43 ± 0.44	65.46 ± 0.44	2.75	145.95
**MB114**	24.27 ± 0.10	42.89 ± 0.36	29.70 ± 0.05	6.42 ± 0.03	1.67 ± 0.02	48.09 ± 0.07	14.12 ± 0.01	36.12 ± 0.07	63.46 ± 0.07	3.41	140.21
**MB115**	26.35 ± 0.17	41.81 ± 0.24	29.57 ± 0.08	6.24 ± 0.25	1.96 ± 0.01	43.18 ± 0.15	19.06 ± 0.06	35.80 ± 0.21	64.46 ± 0.21	2.27	145.49
**MB116**	22.01 ± 0.11	46.93 ± 0.36	29.22 ± 0.05	6.17 ± 0.03	2.30 ± 0.01	47.46 ± 0.02	14.86 ± 0.01	35.38 ± 0.03	64.46 ± 0.03	3.19	141.79

**MB117**	24.94 ± 0.13	45.78 ± 0.21	28.85 ± 0.03	7.17 ± 0.02	2.25 ± 0.01	45.83 ± 0.04	15.90 ± 0.02	36.02 ± 0.04	63.46 ± 0.04	2.88	141.61
**MB118**	26.22 ± 0.19	40.63 ± 0.32	29.72 ± 0.01	5.68 ± 0.03	2.14 ± 0.03	47.87 ± 0.02	14.60 ± 0.01	35.40 ± 0.04	64.46 ± 0.04	3.28	141.66
**MB119**	26.51 ± 0.08	39.77 ± 0.29	29.01 ± 0.03	5.96 ± 0.03	1.97 ± 0.02	48.83 ± 0.01	14.24 ± 0.00	34.97 ± 0.02	65.46 ± 0.02	3.43	142.33
**MB120**	24.79 ± 0.20	43.56 ± 0.14	29.51 ± 0.10	7.12 ± 0.11	2.02 ± 0.02	45.37 ± 0.03	15.99 ± 0.00	36.63 ± 0.02	63.46 ± 0.02	2.84	140.72
**MB121**	24.73 ± 0.20	45.09 ± 0.39	29.07 ± 0.07	8.61 ± 0.32	1.89 ± 0.02	41.25 ± 0.20	19.18 ± 0.09	37.68 ± 0.27	62.46 ± 0.27	2.15	141.94
**MB122**	25.41 ± 0.07	40.33 ± 0.33	29.02 ± 0.02	7.51 ± 0.05	2.38 ± 0.01	46.65 ± 0.03	14.44 ± 0.02	36.54 ± 0.04	63.46 ± 0.04	3.23	138.99
**MB123**	25.14 ± 0.13	40.85 ± 0.10	29.00 ± 0.11	6.33 ± 0.15	2.26 ± 0.00	47.82 ± 0.15	14.60 ± 0.05	35.33 ± 0.20	64.46 ± 0.20	3.28	141.69
**MB124**	24.76 ± 0.06	40.21 ± 0.09	29.71 ± 0.05	6.15 ± 0.05	1.66 ± 0.03	46.86 ± 0.09	15.61 ± 0.09	35.87 ± 0.08	64.46 ± 0.08	3.00	142.22
**MB125**	27.58 ± 0.21	36.84 ± 0.15	29.23 ± 0.05	6.52 ± 0.05	2.28 ± 0.02	46.84 ± 0.10	15.13 ± 0.05	35.75 ± 0.05	64.46 ± 0.05	3.10	141.35
**MB126**	23.86 ± 0.11	44.69 ± 0.19	29.40 ± 0.07	6.34 ± 0.11	1.65 ± 0.02	46.64 ± 0.11	15.98 ± 0.07	35.74 ± 0.07	64.46 ± 0.07	2.92	142.85
**MB127**	25.18 ± 0.04	41.54 ± 0.10	29.06 ± 0.05	7.06 ± 0.01	2.26 ± 0.02	44.86 ± 0.03	16.76 ± 0.09	36.12 ± 0.04	63.46 ± 0.04	2.68	142.25
**MB128**	24.95 ± 0.05	41.29 ± 0.17	28.57 ± 0.04	6.57 ± 0.06	2.34 ± 0.01	48.15 ± 0.07	14.38 ± 0.05	35.14 ± 0.10	64.46 ± 0.10	3.35	141.76
**MB129**	24.50 ± 0.07	43.72 ± 0.38	28.45 ± 0.04	6.81 ± 0.02	2.29 ± 0.01	47.92 ± 0.04	14.53 ± 0.03	35.26 ± 0.03	64.46 ± 0.03	3.30	141.72
**MB130**	26.04 ± 0.22	41.20 ± 0.06	28.89 ± 0.24	7.98 ± 0.89	2.40 ± 0.03	46.08 ± 0.47	14.65 ± 0.16	36.87 ± 0.65	63.46 ± 0.65	3.15	138.51
**MB131**	25.71 ± 0.05	39.55 ± 0.44	28.75 ± 0.07	5.95 ± 0.08	1.92 ± 0.01	48.32 ± 0.01	15.06 ± 0.02	34.70 ± 0.03	65.46 ± 0.03	3.21	143.75
**MB132**	25.58 ± 0.08	40.16 ± 0.18	28.30 ± 0.04	6.95 ± 0.01	2.08 ± 0.01	46.59 ± 0.03	16.08 ± 0.02	35.25 ± 0.04	64.46 ± 0.04	2.90	143.49
**MB133**	26.39 ± 0.18	37.46 ± 0.44	28.65 ± 0.05	6.56 ± 0.04	2.60 ± 0.02	48.42 ± 0.08	13.78 ± 0.02	35.20 ± 0.09	64.46 ± 0.09	3.51	140.77
**MB134**	25.14 ± 0.12	43.25 ± 0.26	29.06 ± 0.04	7.16 ± 0.02	1.84 ± 0.02	46.94 ± 0.05	15.00 ± 0.03	36.22 ± 0.06	63.46 ± 0.06	3.13	140.72
**MB135**	24.73 ± 0.20	44.43 ± 0.18	29.62 ± 0.02	6.66 ± 0.01	2.71 ± 0.01	45.69 ± 0.04	15.32 ± 0.03	36.28 ± 0.02	63.46 ± 0.02	2.98	140.06
**MB136**	24.08 ± 0.09	43.36 ± 0.35	29.71 ± 0.02	6.41 ± 0.01	2.10 ± 0.03	47.10 ± 0.02	14.69 ± 0.00	36.11 ± 0.01	63.46 ± 0.01	3.21	140.37
**Range**	22.01-28.96	32.62-49.03	27.71-31.94	1.62-8.61	1.62-3.15	37.96-50.71	13.78-24.89	31.47-37.68	62.32-68.53	1.53-3.51	138.51-154.98
**Total mean**	25.69	40.85	29.96	4.27	2.30	46.36	17.10	34.30	65.77	2.76	146.33
**Total SD**	1.24	3.00	0.74	1.59	0.36	2.32	2.04	1.69	1.35	0.39	3.66
**Total CV**	4.82	7.34	2.46	37.13	15.46	5.00	11.91	4.00	2.57	14.32	2.50
**p-value**	<0.0001	<0.0001	<0.0001	<0.0001	<0.0001	<0.0001	<0.0001	0.01	0.01	0.001	0.01

Table S3. Factor loadings and contributions of variables in the first six principal components.

	PC1		PC2		PC3		PC4		PC5		PC6	
**Parameter**	**FL**	**%**	**FL**	**%**	**FL**	**%**	**FL**	**%**	**FL**	**%**	**FL**	**%**
Vitexin	0.44	4.95	-0.24	1.72	0.54	14.77	0.58	18.05	-0.24	4.57	0.04	0.16
Isovitexin	0.44	4.95	-0.23	1.62	0.55	15.47	0.57	17.81	-0.23	4.01	0.03	0.07
Total protein	0.17	0.76	-0.23	1.49	-0.37	6.95	0.04	0.11	-0.32	7.94	-0.51	25.50
Total starch	-0.27	1.88	0.20	1.22	0.56	16.13	0.24	3.14	0.42	13.43	0.05	0.26
Palmitic acid	0.26	1.75	-0.18	0.91	-0.40	8.16	0.13	0.95	0.11	0.85	0.76	55.88
Stearic acid	-0.47	5.85	0.81	19.04	0.19	1.90	0.03	0.06	-0.20	3.12	-0.21	4.19
Oleic acid	0.07	0.15	0.22	1.37	-0.15	1.08	0.10	0.51	0.74	41.95	-0.22	4.88
Linoleic acid	-0.04	0.04	-0.52	8.05	0.50	12.81	-0.67	24.14	-0.06	0.24	0.05	0.26
Linolenic acid	0.31	2.49	-0.01	0.00	-0.55	15.48	0.67	24.25	0.05	0.21	-0.13	1.68
TSFA	-0.41	4.47	0.85	21.10	0.01	0.00	0.11	0.67	-0.18	2.46	0.17	2.77
TUFA	0.41	4.47	-0.85	21.09	-0.01	0.00	-0.11	0.67	0.18	2.46	-0.17	2.77
Total saponin	0.52	7.11	0.30	2.58	0.31	4.83	0.02	0.03	0.47	17.04	-0.11	1.15
Total phenol	0.71	13.31	0.38	4.25	0.06	0.17	-0.11	0.62	-0.03	0.06	-0.01	0.01
$DPPH^{\bullet}$ scavenging activity	0.76	14.99	0.51	7.51	0.13	0.86	-0.22	2.60	-0.07	0.35	-0.03	0.09
$ABTS^{\bullet+}$ scavenging activity	0.76	15.02	0.40	4.74	-0.03	0.05	-0.27	3.85	-0.10	0.74	0.05	0.23
Ferric reducing antioxidant power	0.83	17.82	0.34	3.32	-0.16	1.33	-0.22	2.56	-0.09	0.57	0.03	0.10
Eigenvalue	3.83		3.41		1.97		1.84		1.30		1.02	
Variability (%)	23.95		21.30		12.32		11.53		8.11		6.39	
Cumulative variance (%)	23.95		45.25		57.57		69.09		77.20		83.60	
